# The same video game in 2D, 3D or virtual reality – How does technology impact game evaluation and brand placements?

**DOI:** 10.1371/journal.pone.0200724

**Published:** 2018-07-20

**Authors:** Johanna Roettl, Ralf Terlutter

**Affiliations:** Department of Marketing & International Management, Alpen-Adria-Universität Klagenfurt, Klagenfurt, Austria; Universita Cattolica del Sacro Cuore, ITALY

## Abstract

Video game technology is changing from 2D to 3D and virtual reality (VR) graphics. In this research, we analyze how an identical video game that is either played in a 2D, stereoscopic 3D or Head-Mounted-Display (HMD) VR version is experienced by the players, and how brands that are placed in the video game are affected. The game related variables, which are analyzed, are presence, attitude towards the video game and arousal while playing the video game. Brand placement related variables are attitude towards the placed brands and memory (recall and recognition) for the placed brands. 237 players took part in the main study and played a jump’n’run game consisting of three levels. Results indicate that presence was higher in the HMD VR than in the stereoscopic 3D than in the 2D video game, but neither arousal nor attitude towards the video game differed. Memory for the placed brands was lower in the HMD VR than in the stereoscopic 3D than in the 2D video game, whereas attitudes towards the brands were not affected. A post hoc study (n = 53) shows that cognitive load was highest in the VR game, and lowest in the 3D game. Subjects reported higher levels of dizziness and motion-sickness in the VR game than in the 3D and in the 2D game. Limitations are addressed and implications for researchers, marketers and video game developers are outlined.

## Introduction

The video game industry is one of the fastest-growing industries. The global value for the video games market is expected to grow from almost USD 71 billion in 2015 to about USD 90 billion in 2020 [1–3]. At least one person in more than 60% of US American households plays video games on a regular basis, doing so for at least 3 hours per week, and 65% of US American households own at least one device which is capable of playing video games [4]. Similar usage data can be found in Europe or Asia. For instance, 40% of all Eastern Europeans play video games; in Germany, about one in two plays video games occasionally [2,5,6]. In Southeast Asia, every fifth person plays video games, and in China, almost every third person is a video game player [5,7]. In the US, the most popular game genres in 2016 were shooter games (27.5%), followed by action (22.5%), role-playing (12.9%) and sport games (11.7%) [8].

Video games create a virtual reality in which the individual plays the game. Virtual reality is understood as an environment that is created by a computer or other media and in which the user has a feeling of being present in the environment [9]. Over the last few years, there has been a technological change in the video game sector. Instead of a conventional two-dimensional (2D) virtual environment, many video games can be played in a stereoscopic three-dimensional (3D) or even in a Head-Mounted-Display (HMD VR) based virtual reality environment. A 3D environment is deemed to be more realistic and vivid than a 2D environment [10–14]. HMD VR aims at depicting an environment that is even closer to the real world than a 3D environment. Wearing specific VR goggles, a person’s physical presence is simulated in a virtual 3D environment, and at the same time, the goggles shield the individual from the real physical surroundings during the VR experience [15]. HMD VR in particular is currently expected to bring about major changes for the video game sector (e.g. [16]). 63% of frequent gamers in the USA are familiar with the HMD VR technology [17]. The total worldwide market size for HMD VR and AR (augmented reality) is expected to grow from 6.1 USD billion in 2016 to more than 215 USD billion in 2021 [18]. The worldwide sales revenue for HMD VR video gaming only is expected to increase from 5.2 USD billion in 2016 to 22.9 billion US dollars in 2020 [19]. In 2016, North America and Europe were the two biggest markets for HMD VR video gaming with sales revenues of 1.5 billion USD and 1.9 billion USD, respectively. The most popular HMD VR video game genres are adventure, action and simulation games. In 2016, more than 50% of HMD VR video game players were interested in these genres [20].

One phenomenon that exists in many video games are brand placements and they are also addressed in the current research. Brand placements in video games are a form of advertising in which branded goods or services are featured in the video game (e.g. [21–23]). They are “a combination of advertising and publicity designed to influence the audience by unobtrusively inserting branded products in entertainment programs such that the viewer is unlikely to be aware of the persuasive intent” ([24]; p. 89). For game developers, embedding brands is a method to make the game more reality-like and it is also an important income source that contributes to meeting the production costs [25]. For companies, placements in video games are a method of promoting products or brands by embedding them in the game play. The placing companies typically value the chance of relatively intensive and repeated contacts of the player with the brands, especially if the brands are integrated in the main video game plot (e.g. if the player has to use the brands as part of the game play). They also appreciate that brands or products are promoted in a more unobtrusive way than in traditional advertising (e.g. [26]). The most relevant variables for marketers are memory for the placed brands as well as attitudes towards the placed brands [27].

The present study investigates how the game play experience is affected, depending on whether players play an identical video game in 2D, stereoscopic 3D (in the following just “3D”) or Head-Mounted-Display VR (“VR”). In addition, the study analyzes how brand placements in the video games are affected and whether they gain or suffer from advanced technology. By shedding light on the evaluation of 2D, 3D and VR video games and the brands placed therein, the study yields important insights for researchers, video game producers as well as marketers who want to promote their brands via placements in video games.

## Theoretical background

### 2D, stereoscopic 3D and HMD virtual reality video games

In comparison to the conventional 2D technology, which does not give depth to the objects, 3D and VR technology offer additional experiences for the user. The technologies allow for the perception of spatial depth on the screen. In the 3D technology, this is realized by using stereoscopic displays, which creates spatial depth off the screen with 3D pop-up visualizations, so that some objects within the game appear closer to the player and seem touchable, the usage of 3D-capable screens or the usage of dedicated 3D-capable glasses. 3D technology can be more realistic, immersive and allows e.g. improved eye-hand coordination, as compared to the 2D technology experience that lacks depth perception. One of the first 3D home video game devices was Tomytronic 3D in which 3D was simulated using two LCD panels, developed by a Japanese toy maker in 1982. Other early home devices were developed by e.g. Nintendo in 1995 [28,29].

In comparison to the 3D technology, HMD VR tries to deliver an even stronger feeling of being in the world or in the moment [30]. VR is a computer-simulated reality, where the player immerses himself/herself in a fictive 3D world by using a special head-mounted display (HMD), which is a headset that shows visual effects directly in front of the player’s eyes (e.g. Google Cardboard, Oculus Rift, Samsung Gear VR). In VR, the player can interact with and in the environment and is sheltered from the outside world as compared to Augmented Reality (AR) (e.g. Google Glass), where the visuals can be projected on glasses, too, but the player is not sheltered from the outside world) [9,31–33]. In VR the user can move through and experience the game world while thinking that he/she is truly somewhere else. Hence, a VR video game might offer the possibility to be more realistic than a game displaying a 2D or 3D condition [34–36]. Arguably, the first VR headset with goggles was developed in the Mid-Eighties by VPL research and Jaron Lanier. Nowadays, many different VR types and headsets exist. One of the least expensive open source models, developed for Android smartphones, is Google Cardboard. Google Cardboard can be made by the user him- or herself or is sold at prices between 5 USD and 20 USD [30,37]. A more sophisticated device is the Oculus Rift that is currently sold at about 399 USD. Other well-known technologies include the Microsoft HoloLens, a holographic computer, where the user can engage with the digital content and interact with holograms in the world around him or her [38], the PlayStation VR [39], HTC’s Vive, another content streaming headset [40] or the Samsung Gear VR [30].

However, the quality of 3D technologies [13,41,42] as well as VR technologies [35,36] have been debated critically and researchers have demonstrated a range of negative effects, too, e.g. discomfort, eye fatigue, dizziness, headache, disorientation or motion-sickness when using these technologies. Furthermore, VR users might become socially isolated when using head-mounted displays, as a consequence of locking their eyes and ears into a fictional video game generated world for longer periods of time [36,43,44].

### Presence in the 2D, stereoscopic 3D and HMD VR video game

The concept of presence in virtual environments has received a lot of attention during the last decades, especially with the rise of interactive technologies in the 90s, and has been debated from different perspectives (e.g. [31,45,46]). Presence in a virtual environment can be described as the sense of being in a virtually mediated location instead of being in the real location (the place the person is actually located in) [47]. The sense of presence plays an important role in linking perceptions, intentions and actions of an individual in the environment (e.g. [48–52]). High levels of presence in a virtual environment allow the subject to put his / her intentions into action, to monitor the actions and adjust activities if needed. The subject can adapt the own action to the environment [52].

The level of presence seems to be of special interest for the comparison of different technological environments and in the game context. The question is how deeply participants are immersed or “inside” the game [53,54]. Kim and Biocca [55] speak of “departure”, which describes the feeling of not being in the physical environment anymore and “arrival”, which describes the feeling of being within in the mediated environment. Especially in the VR environment, and to a lesser extent in the 3D environment, game players are likely more immersed in the mediated environment (“arrival”) and perceive less of the physical environment (“departure”). If elements of the 3D or VR game play are seemingly touchable as they appear to be around the player, the player probably pays more attention to the 3D effects of the mediated environment, hence the level of immersion and presence should be increased. In the VR video game, wearing the goggles, the player is even sealed off from all visual stimuli around him/her in the physical environment. Hence, there will be an even higher level of “departure” than in 3D. The player will be more immersed and absorbed in the mediated environment, as the stimuli from the mediated environment are practically the only stimuli he/she receives while playing the game, leading to a higher level of “arrival”.

We therefore expect that presence should be higher in the VR than in the 3D than in the 2D video game.

“H1: Presence will be highest in the VR video game, lower in 3D and lowest in the 2D video game.”

### Arousal while playing the video game

Arousal can be defined as stimulation, alertness or activation and is a process, which initiates behavior [56]. According to Bolls et al. [57] arousal “indicates the level of activation associated with the emotional response and ranges from very excited or energized at one extreme to very calm or sleepy at the other” ([56]; p. 629). Measuring arousal in different media formats, such as 3D or VR, has become a common practice in research settings (e.g. [58–61]). Video game players will experience arousal, depending on, for instance, how exiting or involving the playing experience is. Levels of arousal that are too low might lead to boredom and reduced attention directed towards the game. In contrast, if the gamer experiences too much arousal, attention can also be diverted. Hence, the level of arousal elicited by a video game is an important variable [62]. According to previously conducted research, games which are played with 3D or VR can cause a higher arousal than games played with a simpler technology, such as 2D (e.g. [58,63–65]). Thus, we expect a higher arousal in the VR condition than in the 3D than in the 2D condition.

“H2: Arousal will be highest in the VR video game, lower in the 3D and lowest in the 2D video game.”

### Attitude towards the video game

Attitude towards the game in this research is defined as the overall evaluation of the game played. Attitudes are a composite of feelings and beliefs as well as behavioral intentions toward an object [66]. The components are highly interdependent and influence each other. When playing a computer game, individuals will like the game more or less and evaluate it more or less positively or negatively. Attitude towards the game is an important variable for game producers as it determines to a large extent how the player will react to the object, e.g. how much time the player is willing to devote to the game, whether or not he/she replays the game or recommends it to somebody else. Attitudes toward the game are also important because they impact the brands that are embedded in the computer games [67,68].

As outlined above, 3D and VR offer additional technological features (e.g. higher immersion) that may allow for a better attitude towards the game. On the other hand, negative aspects are also related to the technological enhancements (e.g. higher visual fatigue, dizziness), which are likely to impair attitudes towards the game. Since the literature is contradictory with regard to whether the positive or negative aspects related to the technology enhancement dominate, and since no research has examined the attitude towards the game when playing the game either in a 2D or in a 3D or in a VR condition, hence no empirical evidence is available so far, we investigate the impact of the technology on attitude towards the game and formulate the following research question:

“RQ1: Do attitudes toward the game differ in the 2D, stereoscopic 3D and HMD VR condition?”

### Attitude towards the brands placed in the video game

As outlined above, brand placements play an important role in video games. Though previous research has analyzed several factors that influence the recipients’ attitude towards brands placed in computer games, such as game involvement [69], enjoyment and attitude toward the game [70], or brand prominence [71], surprisingly little research has addressed how different delivery modes such as 2D, 3D, or VR might impact the brands placed in video games. Drawing from related research fields, mainly from advertising and product presentation on websites, there is some indication that brands might benefit from 3D as compared to 2D presentation. Li et al. [72] found that ‘flat’ 3D advertisements on websites generate more positive brand attitudes than 2D advertisements. Flat 3D means that the product representation is 2D, but that users can rotate products, animate their functions and features or can zoom in or out for inspection. These findings are also consistent with the study conducted by Choi and Tylor [73], which shows that flat 3D brand representations (by moving, zooming and rotating the object) on websites leads partly to a higher brand attitude than 2D brand representations (static pictures). However, this was only the case for a geometric product (watch) but not for the tested material product (jacket) [73]. Debbabi et al. [74] report similar findings, since the brand attitude was more positive for flat 3D Internet-based advertisements than for 2D ones. According to Lee et al. [75], consumers’ brand attitudes were more responsive and were held with greater confidence for flat 3D visualized products on an interactive website than for 2D products on a website that was static. Kerrebroeck et al. [76] demonstrate that attitude toward the ad, attitude toward the brand and purchase intentions were higher in the case of VR versus 2D. In their experiment participants watched a video either in a 2D condition on a mobile phone or in a VR condition on a HMD based Google Cardboard-type device.

To the best of the authors’ knowledge, no study has addressed this question in the context of 2D, stereoscopic 3D and HMD VR video games. Hence, several studies have shown that an enhanced technology might influence the attitude towards the brand positively. We therefore derive the following hypothesis:

“H3: Attitude toward the placed brands will be more positive in the VR video game compared to the 3D, and it will be least positive in the 2D video game.”

### Memory for the placed brands

Recall and recognition are the most common measures to examine the memory of brand placements (e.g. [25,27,77–79]). Recall is the ability of a person to retrieve a brand name correctly from memory without any mention of other brand names or the product class. Recognition is the ability to remember that there exists past exposure to the brand and it is usually measured by using aided memory based techniques, e.g. where brands are listed and the person can choose the brand/s which he/she has recognized [80,81]. One model that may explain effects of technology enhancement on the memory for brand placements in computer games is the limited capacity model of motivated mediated message processing [82]. The model assumes that an individual’s attentional capacity and his/her ability to process information cognitively is limited. The cognitive capacity, which is used to perform a primary task, cannot simultaneously be used to accomplish a secondary task. When playing video games, the game play is the primary task because the player primarily focuses his/her attention on those aspects which are relevant for a successful game play. While the player focuses his/her attention on the primary task, fewer cognitive resources are available for secondary tasks, such as processing embedded advertisements [82–85]. The more attentional capacity is needed to play the video game, the less capacity will be left for processing the information about the placed brands [83,84].

It can be assumed that a 3D and a VR condition require more cognitive resources than a 2D condition. The depth perception in the 3D and the VR environment and the higher complexity of the VR world in general are additional items of information that occupy more cognitive resources. There is also empirical evidence that supports this assumption. Mun et al. [86] demonstrated in cognitive tests that the brain activity of recipients who were exposed to a stereoscopic 3D environment was higher than that of subjects who were exposed to a 2D environment. Furthermore, those who were exposed to the 3D environment needed longer execution times for tasks and paid more attention to the 3D effects than to other areas because of their visual fatigue (exhaustion of the eyes). Yim et al. [87] explored how stereoscopic 3D technology in comparison to a 2D display influences the viewers’ memory of brand names embedded in a soccer game. Results showed that the viewers remembered less brand names in the 3D condition compared to the 2D condition [87]. Other studies also found that subjects have a longer reaction time in 3D conditions than in 2D conditions, since their cognitive load is increased (e.g. [88–90]). Furthermore, in a 3D as well as in a VR environment, the backgrounds are often blurred and the 3D or VR environment can stress the viewers’ eyes, which can also lead to cognitive fatigue and a reduction of attention. Comparing 2D, 3D and 4D movies (3D plus scent), Terlutter et al. [91] found that memory for brand placements suffered in the 3D and 4D condition as compared to the 2D condition, except for one extremely prominent placement that was better memorized in the 3D condition, and they attribute the typically lower memory in 3D and 4D to the greater amount of cognitive resources needed for processing the central movie plot, leaving less resources for processing the brand placements.

Thus, the player has to process more information in a VR and a 3D video game in comparison to a 2D video game and hence more cognitive resources are needed for the game play (the primary task), leaving less cognitive resources for secondary tasks, such as memorizing the brand placements in the video game. This leads us to the following hypothesis:

“H4: Recall and recognition of the brands included in the video game will be lowest in the VR condition, higher in the 3D condition and highest in the 2D condition.”

### Control variables

Skepticism towards advertising, general attitude towards video games, prior video game playing experience and video game literacy serve as control variables.

## Method

In Austria, it is not necessary to go through an Institutional Review Board or an Ethical Committee when performing a study with human participants who are of legal age. The research design and the questionnaire are in line with the Austrian and the EU privacy regulations. The research as well as the questionnaire have been approved by several professors and employees of the University. Subjects have been notified and have been properly instructed about their voluntary participation in an experiment and that their data will be handled strictly confidential by using appropriate tools, instruments, and protocols to secure their privacy. Consenting participants have been informed about the survey verbally and in written form in advance and after filling out the questionnaire. The data was handled in a strictly confidential fashion and anonymously.

### Main study design

In order to address the above proposed hypotheses and research question, a “jump’n’run” video game was designed and developed in a 2D, 3D and VR condition by a professional game designer. The 2D condition was developed with traditional pictures and without depth of the objects. The 3D condition refers to stereoscopic three-dimensional technology, where the objects pop-up with true depth and float off the screen [14]. The VR condition was based on the HMD technology. The game was designed with the software tool Unity 3D. We chose a jump’n’run action game for several reasons: Action video games place a focus on the player’s reflexes as well as on his / her reaction time, the eye-hand coordination is important and players try to accomplish a goal [92]. “Jump’n’run” video games are one of the most common game genres and spatial depth perception is likely to be of relevance for game play. The game consisted of three levels of increasing difficulty. Playing time was between 7 and 10 minutes. In the 2D and 3D condition, the game was played on a large 46-inch, stereoscopic 3D-capable television. Participants who played the game in the 3D condition received special 3D glasses, which allowed the players to experience depth perception. Players of the VR condition wore an Oculus Rift headset. The whole game was accompanied by the same music in all three conditions. Participants played the game by using an Xbox 360 controller for Windows.

In total eight different brands were integrated in the game, each brand appeared in each level (hence each brand appeared three times during game play). The brands belonged to the following product categories: airline, chocolate, bank, energy drink, coffee, fitness club, nachos, and smartphone. All brands were fictitious to avoid confounding effects of previous brand knowledge. The size and presentation type of all brands were kept constant across all three different levels and across all three game conditions. The brands remained on screen until they were collected by the player. One goal that players pursued was to collect as many brands as possible in order to get to the next level. All participants reached the last level. At the end of the game, participants could record their name in a high score list. After playing the game, each participant filled out an electronic questionnaire (see S1 Appendix).

Data was analyzed with SPSS (see S1 Data). Analyses of variance and t-tests were carried out. In order to determine the practical and theoretical relevance of an effect as well as the power of the analyses, effect sizes were estimated for each analysis [93]. Partial eta-squared (η^2^_p_) was used for the effect size measurement for the analysis of variance (ANOVA). It measures the strength of the effect on a continuous field, where η^2^_p_ = 0.01 indicates a small effect, η^2^_p_ = 0.06 indicates a medium effect and η^2^_p_ = 0.14 indicates a strong effect [94–96]. Hedges’ g (g_Hedges_) was used to evaluate the effect of group differences of t-tests. Hedges’ g accounts for different group sizes and also allows for smaller group sizes [97–100]. Values of g_Hedges_ = 0.2 indicate a small effect, g_Hedges_ = 0.5 a medium effect, and g_Hedges_ = 0.8 a large effect. Phi (ϕ) was used to measure effect size for the chi-squared test, whereas ϕ = 0.1 indicates a small effect, ϕ = 0.3 indicates a medium effect and ϕ = 0.5 indicates a large effect [100,101].

### Pre-test

Before conducting the main study, a first pre-test was carried out to develop and to test the video game in all three conditions (n = 10 students). The aim of the pre-test was to ensure that the game was neither too difficult nor too easy to play, that the brand positions were appropriate and not annoying, and that the questionnaire was comprehensible and not too long. None of the participants had any prior video game experiences in 3D or VR. Three subjects played the 2D video game, four people played the 3D video game and three subjects played the VR video game. Minor adaptions were made in the video game and in the questionnaire based on students’ feedback. All ten students were of the opinion that the programed game was of high quality and fun to play, regardless of the technology they had played. None of the students mentioned any concerns regarding playing the game or filling in the questionnaire.

### Participants

237 students from a midsize university in Europe participated in the main study, held in computer labs on the campus. Participants were randomly assigned to one of the three game conditions (2D, 3D, VR). Three participants were excluded from the analysis because of an extremely short answer time and/or inconsistent answer patterns (e.g. flatliners, contradictions), resulting in 234 usable respondents (2D: 79 subjects; 3D: 78; VR: 77). We used a student sample, because most video game players are aged 18–49 and students are in the main target group for a jump’n’run video game like the one at hand [102,103]. Students received extra credit in a course in exchange for their participation; in addition, chocolate bars were given as small incentives as well as the option to win a voucher for a local shopping center. Respondents were between the ages of 18–46, with a mean age of 24.52 years (SD = 4.13). Age distribution did not differ between the three conditions (2D, 3D, VR) (F(2,230) = 2.165; ns, η^2^_p_ = .018). Females (n = 134) were slightly overrepresented in the study in comparison to males (n = 100), but gender distribution did not differ between the three conditions (χ^2^ = 1.826_(df = 2)_, ns, ϕ = .088). 48.3% of the participants played video games at least once per month with an average playing time of 22.29h per month (SD = 33.04). Men (M = 31.26h per month, SD = 40.19) played video games almost three times as often as women (M = 11.34h per month, SD = 15.62) (t = -4.272_(117,205)_, p<0.01, g_Hedges_ = .63), but average monthly playing time did not differ between the three conditions (F(2,157) = .041; ns, η^2^_p_ = .001).

### Measurement of variables

Measurement of the variables was based on existing literature (see S2 Appendix). All interval scaled items had a “no answer” category as an alternative. Demographic data had to be provided (e.g. gender, age, field of study) at the end of the questionnaire. Data were analyzed using SPSS version 22 (IBM Corp, Armonk, NY, USA) (see S1 Data and S2 Data).

Attitude towards the game (α = .864) was measured with six bipolar adjectives, using 7-point semantic differential scales, with the negative adjectives coded 1 and the positive adjectives coded 7. The adjectives were: unappealing-appealing, unpleasant-pleasant, dull-dynamic, unattractive-attractive, not enjoyable-enjoyable, and depressing-refreshing [104]. The mean value of the items was calculated and used for further analyses.

Attitude towards the brand (α_nachos_ = .916; α_chocolate_bar_ = .932; α_smartphone_ = .930; α_energy_drink_ = .951): In order to avoid an overly long questionnaire, attitude toward the brand was measured for only four out of the eight brands that were placed in the game. Subjects were asked to evaluate the brands based on the following six items, using a 7-point scale: this is a bad / good product, I feel negative / positive toward the product, the product is awful / nice, the product is unpleasant / pleasant, the product is unattractive / attractive, I disapprove / approve of the product. The questions were adapted from Shamdasani et al. [105]. The mean value of the items was calculated and used for further analyses.

Arousal (α = .846) was measured immediately after playing the video game. Subjects were asked to indicate their perceived level of arousal based on three bipolar adjectives and on a 7-point semantic differential scale, hence, a self-reported arousal measurement was used. The items read excited-calm, stimulated-relaxed and alerted-soothed [56]. The mean value of the items was calculated and used for further analyses.

Presence was assessed by asking about the level of agreement to the following statement: “I was totally absorbed in what I was doing” [106]. The answer scale ranged from 1 (low agreement) to 7 (full agreement). Hence, the current research applied a single item measurement of presence. A single item measurement was chosen for practical reasons to avoid an overlong questionnaire. In addition, the level of absorption in what somebody is doing in an environment is an important indicator for the individuals’ presence in that environment. If individuals’ presence in an environment is high, they are able to adapt their actions to the environment [52] and individuals do not necessarily perceive the state of being in the virtual environment [53].

Brand recall was measured by asking the participants to write down the remembered brands, which they had seen while playing the game.

To measure brand recognition each of the eight brands that had appeared in the game was presented along with two mock brands from the same product category which had not appeared in the game. Participants had the possibility to choose “others”, if they felt they could not remember any of the three brands. The eight brands with their respective mock brands were presented one after the other, and participants could only tick one option. This option was either the correct brand (true memory) or not (i.e. false or no memory). If the participants just guessed, there would be a 25% probability of choosing the brand that appeared in the game for each brand category, which would be same for all conditions. We deliberately chose to measure recognition for each brand one after the other, instead of presenting a list with all appearing brands and the mock brands at once. Our measurement avoids the problem that is related to presenting a long list of all appearing brands and mock brands at once, namely that memory is easily overestimated if participants just start ticking many alternatives (and by doing so have the chance to hit the correct brands, too, even though they do not recognize them). However, our measurement does not allow for a recognition sensitivity test as suggested by e.g. Grier [107]. Brand logos were presented in random order for each participant to avoid order and context effects. 1 was coded if a subject named or ticked the correct brand, otherwise 0 was coded.

For both, recall and recognition, the named or ticked brands were added up to an 8-point sum scale for each participant.

#### Control variables

Skepticism towards advertising (α = .899) was examined according to Obermiller & Spangenberg [108] by using the following nine items: we can depend on getting the truth in most advertising; advertising's aim is to inform the consumer; I believe advertising is informative; advertising is generally truthful; advertising is a reliable source of information about the quality and performance of products; advertising is truth well told; in general, advertising presents a true picture of the product being advertised; I feel I've been accurately informed after viewing most advertisements; most advertising provides consumers with essential information. The answer scale ranged from 1 (low agreement) to 7 (full agreement). Hence, low values of the items indicate high levels of skepticism. The mean value of the items was calculated and used for further analyses.

General attitude towards video games was measured by asking the subjects “In general, what kind of feelings do you have toward video games?”, where 1 was “very negative” and 7 was “very positive” (adapted from Porter & Donthu) [109].

Prior video game experience was investigated by asking the following question: “How much experience with games of this type (jump’n’run games), such as you have just played, do you have?” The answer scale ranged from 1 (no experience at all) to 7 (a lot of experience).

Video game literacy was measured with the following question: “How good are your skills in relation to video games?”, where the answer scale ranged from 1 (no skills at all) to 7 (very good skills).

## Results of the main study

Before the hypotheses and research question were addressed, ANOVAs were conducted with the three groups (2D, 3D, VR) as independent variable and the control variables skepticism towards advertising, general attitude towards video games, prior video game playing experience and video game literacy as dependent variables. Analyses revealed that the three groups did not differ significantly in all four control variables (skepticism towards advertising: F(2,231) = .127, ns, η^2^_p_ = .001; general attitude towards video games: F(2,230) = .741, ns, η^2^_p_ = .006; prior video game playing experience: F(2,231) = .186, ns, η^2^_p_ = .002; and video games literacy: F(2,231) = .338, ns, η^2^_p_ = .003. Additionally, gender differences were checked. As could be expected, significant gender differences with regard to prior video games experiences with jump’n’run games could be revealed, with males being more experienced than females (t = -5.835_(230.079)_, p<0.01, g_Hedges_ = .748).

### Presence in the 2D, 3D and VR video game

H1 postulates a higher level of presence in the VR condition than in the 3D condition than in the 2D condition. An ANOVA with the three different technologies as independent variable and presence as dependent variable revealed that the three groups differed significantly in their presence (F(2,228) = 5.104, p<0.01, η^2^_p_ = .043). Results show that the mean was lowest in the 2D condition (M = 5.28; SD = 1.71), followed by the mean of the 3D condition (M = 5.63; SD = 1.38). The mean was highest in the VR condition (M = 6.03, SD = 1.21). Contrast tests show that significant differences were found between the 2D and VR condition (t = 3.13_(139.19)_, p < .01, g_Hedges_ = .499). The differences between the 2D and 3D condition (t = -1.40_(147.24)_, ns, g_Hedges_ = .225) as well as between the 3D and VR condition (t = -1.87_(147.95)_, .063, g_Hedges_ = .300) were as expected, but were not significant. Hypothesis H1 was partly supported by the data.

### Arousal while playing the video game

Regarding the second hypothesis that arousal will be higher in the VR video game than in the 3D than in the 2D video game, an ANOVA with technology (2D, 3D, VR) as independent variable and arousal as dependent variable revealed no significant differences between the three different technological conditions (F(2,228) = .984, ns, η^2^_p_ = .009). Players in all three technology groups were moderately aroused (2D: M = 4.18, SD = 1.57; 3D: M = 4.25, SD = 1.60; VR: M = 3.90; SD = 1.63). H2 is rejected by the data.

### Attitude towards the video game

In order to answer the research question whether attitudes toward the game differ in the 2D, 3D and VR condition, an ANOVA with technology (2D, 3D, VR) as independent variable and attitude towards the game as dependent variable was carried out. The analysis showed no significant differences between the three technologies (F(2,226) = .365, ns, η^2^_p_ = .003). The mean values in all three technology groups were relatively high, indicating that the players liked the video game, regardless of the technology they had played (2D: M = 5.08, SD = 1.09; 3D: M = 5.24, SD = 1.16; VR: M = 5.16; SD = 1.08).

### Attitude towards the brands placed in the video game

To address the third hypothesis that attitude toward the placed brands will be higher in the VR video game than in the 3D than in the 2D video game, four consecutive ANOVAs, one for each brand in the four product categories, were performed (independent variable = technology, dependent variable = attitude towards the brand). Results of the ANOVAs revealed that the attitude towards the placed brands did not differ among the three technology groups, for any of the four brands (nachos: F(2,224) = .437, ns, η^2^_p_ = .004; chocolate bar: F(2,226) = 1.340, ns, η^2^_p_ = .012; smartphone: F(2,225) = 1.186, ns, η^2^_p_ = .010; energy drink: F(2,227) = .748, ns, η^2^_p_ = .007).

### Memory for the placed brands

Hypothesis H4 expected that recall and recognition of the brands included in the video game will be lower in the VR condition than in the 3D condition than in the 2D condition.

#### Recall

An ANOVA with technology as independent variable and recall as dependent variable revealed significant differences among the three groups (F(2,231) = 8.514, p < .01, η^2^_p_ = .069). As expected, subjects in the VR condition had the lowest recall of brands (M = .61, SD = .96), subjects in the 3D condition had a higher recall (M = 1.06, SD = 1.33) and subjects in the 2D condition had the highest recall (M = 1.52, SD = 1.72). Contrast tests indicate that the differences between 2D and VR (t = -4.09_(123.24)_, p < .01, g_Hedges_ = .651) as well as between 3D and VR (t = 2.43_(140.18)_, p = .016, g_Hedges_ = .388) were significant. The difference between 2D and 3D (t = 1.86_(146.86)_, p = .065, g_Hedges_ = .299) was significant only on the 10% level. Gender did not affect brand recall (t = -1.923_(185,788)_, ns, g_Hedges_ = .262).

The above mean values indicate that, overall, brand recall appeared to be low. In the 2D condition 32 participants could not recall any brand. In the 3D condition 39 students did not recall any brand, whereas in the VR condition 50 participants memorized none of the brands.

#### Recognition

An ANOVA revealed that the three technology groups differed significantly in their brand recognition (F(2,231) = 14.571, p < .01, η^2^_p_ = .112). Similar to the results for brand recall, in the VR condition, subjects recognized fewest brands (M = 3.42, SD = 1.51), subjects in the 3D condition recognized more brands (M = 4.23, SD = 1.77) and subjects in the 2D condition recognized the highest number of brands (M = 4.91, SD = 1.90). Contrast tests showed that all pairwise differences were significant (2D and 3D: t = 2.33_(154.47)_, p = .021, g_Hedges_ = .371; 2D and VR: t = -5.46_(148.07)_, p < .01, g_Hedges_ = .869; 3D and VR: t = -3.09_(149.91)_, p < .01, g_Hedges_ = .493). Gender did not affect brand recognition (t = -.272_(232)_, ns, g_Hedges_ = .038). The values of brand recognition were much higher than the values for brand recall, which could be expected. Over all three conditions, only four participants did not recognize any brand.

Summarizing, the data lends support to our expectation that memory for placed brands will be lower in the VR than in the 3D than in the 2D condition (see S3 Appendix). H4 on brand recall and brand recognition receives substantial support.

## Post hoc study on cognitive load and physical reactions while playing the video game

A post hoc study was carried out in order to gain more insights into the cognitive load and some physical reactions (dizziness and motion-sickness) while playing the video game. In addition, the aim was to confirm the memory results of the main study.

Fifty-three students participated in the post hoc study. The same video game as in the main study was used and played in either 2D, stereoscopic 3D or HMD VR. 19 students played the 2D video game, 19 students played the video game in 3D and 15 students played the video game in the VR condition. 37 participants were female, 16 were male.

### Cognitive load

Cognitive load was measured to test our assumption made in the theoretical part that participants playing the video game in the 3D and VR condition need more cognitive capacity for playing the game, as compared to participants in the 2D condition. Cognitive load refers to the total amount of mental effort that is used in the working memory when performing a learning task, it is “the manner in which cognitive resources are focused and used during learning” ([110]; p. 294). To measure cognitive load, participants in all conditions were asked to memorize the same 8-digit number prior to playing the video game [111]. After playing the video game, participants were asked to recall as many digits as possible of the number they had been given. A lower number of digits that can be memorized and recalled result from higher cognitive load while playing the game.

The results showed that the mean of correctly recalled digits was indeed lowest in the VR condition (M = 3.47; S = 1.06). However, other than expected, the mean of the 2D condition (M = 4.00, SD = 1.56) was lower than the mean in the 3D condition (M = 4.42, SD = 1.56). Hence, cognitive load while playing the video game appeared to be highest in the VR condition and lowest in the 3D condition. While an ANOVA with the three conditions as independent variable did not reveal significant differences between the three groups (F (2,50) = 2.50, ns, η^2^_p_ = .091), post hoc t-tests between two groups revealed that the mean of the number of correctly recalled digits in in the 3D and VR condition differed significantly (t = -2.236_(5)_, p = .03, g_Hedges_ = .696). There were no differences between male and female participants (t = .233_(51)_, ns, g_Hedges_ = .074).

### Brand memory (recall and recognition)

Recall and recognition of brands were measured in the same way as in the main study. The results of the post hoc study confirmed the findings of the main study. With regard to recall, the three technologies differed significantly (Recall: F(2,50) = 5.237, p < .01, η^2^_p_ = .181). Recall was lowest in the VR condition (M = .40, SD = .91), followed by the mean in the 3D condition (M = .63, SD = 1.07), and highest in the 2D condition (M = 1.58, SD = 1.30). Significant differences between 2D and 3D (t = 2.609_(50)_, p = .012, g_Hedges_ = -.798) and 2D and VR (t = -3.049_(50)_, p < .01, g_Hedges_ = 1.03) were revealed. The results for brand recognition were similar (F(2,50) = 5.479, p < .01, η^2^_p_ = .180). Recognition was lowest in the VR condition (M = 3.00, SD = 1.73), followed by the 3D condition (M = 4.52, SD = 1.71), and highest in the 2D condition (M = 4.79, SD = 1.55). Post hoc t-tests revealed significant differences between 3D and VR (t = 2.661_(50)_, p < .01, g_Hedges_ = .884) and 2D and VR (t = -3.230_(50)_, p < .01, g_Hedges_ = 1.097). Values for brand recognition were again much higher than values for brand recall.

### Dizziness and motion-sickness while playing

Perceived dizziness and motion sickness while playing the game were also measured. Dizziness was measured with the question “Did you feel dizzy when playing the video game”, motion-sickness with the question “Did you become motion sick while playing the video game?”. The questions were adopted from Jones et al. [112] and Merhi et al. [113]. The answer scale ranged from 1 (not at all) to 7 (very much).

Subjects reported significantly different levels of dizziness that they had experienced during game play (F(2,50) = 10.265, p < .01, η^2^_p_ = .291). Dizziness was significantly higher while playing the VR game (M = 2.60, SD = 1.55) than while playing the 3D game (M = 1.47, SD = .84; t = 2.536_(20.416)_, p = .019, g_Hedges_ = .939) and the 2D game (M = 1.11, SD = .32; t = -3.677_(14.918)_, p < .01). No significant differences were found between playing the 2D and 3D game (t = 1.788_(22.960)_, ns, g_Hedges_ = .566).

Results for motion-sickness were similar (F(2,50) = 12.359, p < .01, η^2^_p_ = .331). Subjects reported significantly higher levels of motion-sickness in the VR game (M = 2.40, SD = 1.64) than in the 3D game (M = 1.05, SD = .229; t = 3.160_(14.434)_, p < .01, g_Hedges_ = 1.229) and the 2D game (M = 1.05, SD = .229; t = -3.160_(14.434)_, p < .01, g_Hedges_ = 1.229). Again, no significant differences were found between 2D and 3D (t = .000_(36.000)_, ns, g_Hedges_ = 0).

## Discussion

This study sheds more light on our understanding of 2D versus 3D versus VR video games by analyzing whether and how the delivery mode of an identical video game in either 2D, 3D, or virtual reality (VR) impacts players’ game evaluation as well as the brands that are placed in the game.

Presence was highest in the VR video game and lowest in the 2D video game, presence in the 3D game was in between. The VR video game, and to a lesser extent the 3D video game, obviously lead to a higher feeling of being in the mediated gaming environment (high levels of “arrival”, as outlined in the beginning) and to a lower perception of the real physical environment (high levels of “departure” [55] as compared to the 2D video game). The latter is probably mostly due to the goggles that individuals wear during game play and that shield them from all visual stimuli of the physical environment. The fact that the difference in presence between the 2D and 3D was not significant, but the difference between 2D and VR was, is an indicator that individuals perceive 2D as quite different to VR, whereas the step from 2D to 3D is perceived as less substantial. Obviously, the VR environment offers more potential for immersion and the feeling of “being there” than the 3D environment. The finding that immersion and absorption between the 2D and 3D condition change to a lesser extent corroborates findings of Williams [22].

The level of arousal that players reported to have experienced while playing the game did not differ between the three technologies. This is an interesting finding. Even though presence and the feeling of “being in the game” is higher in the VR and 3D video games, the players’ level of arousal seems to be comparable, regardless of technology. A recent finding from the movie sector that has analyzed movie viewers’ reactions to a movie that was either aired in 2D or in 3D points into a similar direction. The study found that, even though viewers in the 3D condition rated their experience as more realistic than viewers in the 2D condition, no significant differences were found with regard to movie viewers’ emotional arousal [12].

With reference to the attitude towards the game, no group differences between the 2D, 3D and VR technology occurred. As was outlined in the theoretical portion of this paper, both, the 3D and the VR technology offer additional features that might contribute to a higher liking of the game. Yet, on the other side, the new technologies often come along with negative experiences during usage such as dizziness, headaches, or motion sickness (e.g. [36,43,44]). The post hoc study showed that the VR players also sensed dizziness and motion-sickness while playing the game. It appears that the possible disadvantages of the enhanced technology counterbalance the possible advantages with regard to attitude towards the game, at least in our video game.

As regards how brand placements which are commonly found in video games, are affected by the video game technology, our findings indicate that attitudes towards the brands placed in the video games are not influenced by the technology, but that memory of the brands is. In both, the main study as well as the post hoc study, subjects playing the 2D video game were more likely to recall and recognize brand placements compared to subjects who played the 3D or VR video game. In both studies, the memory was highest in the 2D group and was diminished by the additional technology in the 3D group and further reduced in the VR group.

Thus, the results from both studies clearly indicate that in terms of memory, brand placements suffer by an increase of the video game technology. We expect that performing the primary task of playing the game likely needs more cognitive resources (i.e. higher cognitive load) in the VR and the 3D video game as compared to the 2D video game, hence, these cognitive resources are no longer available for performing secondary tasks, such as processing and memorizing the brand placements in the game. Our post hoc study on cognitive load while playing the video game lends at least some support to our interpretation. The study showed that cognitive load was highest in the VR playing condition. However, cognitive load was lower in the 3D than in 2D condition, which was contrary to expectations (though differences were not significant). Obviously, additional research is needed. Our interpretation of the results points in the same direction as earlier research in the field of neuroscience, which indicates that the VR technology requires more cognitive capacity than the 3D and that 3D requires more than 2D technology (e.g. [41,86,88–90]). The findings that memory for the brand placements is lower in the VR than in the 3D condition indicate that the VR technology probably needs even more cognitive capacity for the primary task than the 3D technology. In the VR video game, the player has to deal additionally with a 3D-world in which the player can move through and experience the world individually. While immersed in a fictional world and believing him- or herself to be somewhere else, the player is likely even more focused on the game itself and has less cognitive capacity left to memorize the integrated brands.

Another explanation for the lower memory in the VR and 3D video playing condition might be the higher levels of dizziness and motion-sickness that players experienced during game play, as compared to players in the 2D video game. These negative physiological reactions might have distracted individuals from the brands in the game, leading to lower memory for brands in these conditions.

The fact that brand recall was relatively low in all three gaming conditions deserves some elaboration. While recognition only requires a simple familiarity decision about whether the stimulus has been encountered before, recall needs active reconstructing of information, i.e. remembering a fact, event or object that is not currently physically present and requires high mental efforts. Hence, it is not surprising that the brand recall values were much lower than the brand recognition values, over all three gaming conditions. The low brand recall indicates that players were distracted from the brand during the time of encoding, which is likely to have impaired subsequent retrieval success of the brand name. As was outlined in the theoretical part above, the players’ primary task was the successful playing of the game, rather than the encoding of the brand names they were confronted with during game play. Obviously, the primary task of the game play bound many cognitive resources that were consequently no longer available for memorizing the brand names. It is not uncommon, however, that brand recall is found to be low in advertising and placement studies, especially if the brands are not placed prominently enough. In a recent study of recall of brands that were placed in Hollywood movies, recall of brand names was also low (e.g., of 26 brands that appeared in one of the movies, movie watchers could only recall between 1 or 2 brands on average). In another movie, recall was even lower [91]. It is very likely that higher levels of brand prominence in a game are needed (e.g. the players in a car racing game drive a specific car brand, the players interact with a brand intensively, etc.) to increase brand recall.

## Implications

The findings of our study are relevant for game developers, marketers and researchers. This study contributes to research and theory in various ways, since it empirically examines the evaluation of video games and the brand placements within the games, by directly comparing players’ reactions to a 2D, stereoscopic 3D and Head-Mounted Display VR video game. Our results indicate that 3D and VR lead to higher presence, i.e. to a pronounced feeling of “being in the game”, but game evaluation did not differ between the 2D, 3D, and VR version. This is an important finding, because it shows that an enhancement in technology to 3D or VR does not necessarily lead to a better game evaluation. The additional depth perception in the 3D environments and the increased presence lead to a higher cognitive load and also come along with negative aspects such as dizziness and eye fatigue that probably impair video game evaluation. Subjects who played the VR game in particular reported higher levels of dizziness and motion-sickness while playing the game. Hence, the fact that video game evaluation was not worse in the 2D condition as compared to the 3D and VR condition indicates that game developers can still be quite successful by continuing to offer “traditional” 2D video games. Game developers of 3D or VR video games need to be aware that the 3D and VR experiences can come along with negative feelings that could possibly harm game evaluation, so they need to develop video games in which the advantages of 3D or VR use clearly outweigh these associated disadvantages.

The research also offers implications for brand placements in 2D, 3D, or VR video games, which are important for both, marketers who want to promote their brands, as well as for game developers who often seek placements to contribute to meeting the production costs and as a means to enhance the perceived reality of the games. According to the results of both our studies, memory for the brands placed is negatively affected by enhancement in technology, i.e., in the VR and in the 3D video game, players remembered a lower number of the brands, as compared to the 2D video game, while attitudes towards the brands were unaffected by the technology. The finding that memory is lower with enhancement in technology while at the same time attitude towards the brands does not benefit is an important finding as it indicates that marketers may stick to 2D video games when they seek to promote their brand via placements in video games. In the VR and in the 3D condition it seems very likely that the player needs more resources for the game play, resources that are then no longer available for the processing of the brands placed in the movie.

## Limitations and Directions for future research

There are some limitations within this study. First, we were able to demonstrate that attitudes towards the game did not differ between the three video game technologies and we assumed that possible advantages related to the new technology (such as higher immersion in the mediated world) are counterbalanced by possible disadvantages of the technologies (such as motion sickness). We measured dizziness and motion-sickness in a post hoc study, but not in the main study. Future studies might want to put a stronger focus on advantages and disadvantages that players associate with the new technologies. We used an easy to play “jump’n’run” game, which is a very common action game genre among players. Further research could examine the influence of 3D and VR video games in different video game genres (e.g. sports games such as football games or car racing games, ego shooter games or arcade) or different sectors (e.g. health area) with different product categories and try to find out the generalizability of the findings to other game types and kinds of games (e.g. video games on social media platforms or mobile devices).

Another field of research that certainly needs additional attention is the level of cognitive load needed to experience the different technologies. We have tried to analyze the cognitive load based on theoretical reasoning and by drawing upon extant studies and we measured cognitive load in a post hoc test. However, cognitive load was not measured in the main study. Future research may focus on cognitive load while subjects are playing a 2D, 3D and VR video game. Future research may also want to apply different measures of cognitive load, e.g. by self-reports of the players’ invested mental effort, by a different dual-task methodology (e.g. [62,114,115]), or by electroencephalography. We also did not measure the players’ visual attention, e.g. by using a reliable eye-tracking device. Moreover, of current interest, a longitudinal study would be interesting to ascertain whether the negative impact of the VR technology on brand memory is due to the newness of and lack of experience with the technology. Furthermore, future studies can analyze the moderating role of prior video game playing experience on the relationships of the variables.

It would also be interesting to investigate familiar brands in the placements instead of unfamiliar brands. According to previous research, players usually memorize brands more easily to which they have been exposed previously [116,117]. In our measurement, each brand that had appeared in the game was presented with three other options at the same time, two mock brands from the same product category and the option “others”, and recognition for the eight brands was measured one after the other. Even though this measurement helps to avoid overestimation of memory because participants cannot simply tick many alternatives in a long list of appearing brands and mock brands (if all brands were presented at the same time), a recognition sensitivity test (e.g. [107]) cannot be applied to our data and future studies may take this into consideration when designing the measurement.

Another limitation of this study is that, although we asked about prior game experience, subjects played the video game only once and did not repeat playing it. While playing the same video game several times, repeated exposure to the same brand placements might influence the brand memory differently in the different technologies [118]. Thus, in future studies, an experiment with repeated game playing could also be conducted.

One aspect that needs additional research is the role of presence while playing the video games. In our research, presence was conceptualized as the level of absorption that subjects perceived while playing the game. However, presence can be understood and conceptualized in different and more elaborated ways, too (e.g. [52]). As stated by Triberti and Riva [52], presence is defined as a cognitive process with the purpose “to locate the Self in a physical space or situation, based on the perceived possibility to act in it” [52]. This sense of presence allows the individual to adapt his / her own actions to the external environment [52]. According to Zahorik and Jenison [119], presence is “tantamount to successfully supported action in the environment*”* ([119]; p. 87), where the environment can be virtual or real and can be near to or far from the person. The term “successfully supported action” means that the environment reacts to the person’s actions in a way that is “commensurate with the response that would be made by the real-world environment in which our perceptual systems have evolved” ([119]; p. 87), leading to high levels of presence [119]. Lombard and Ditton [53] define presence as perceptual illusion of non-mediation. The term “perceptual” refers to real time responses or feelings towards an object or entity in a person’s environment and involves real time responses from the individual’s affective and cognitive processing systems or the individual’s sensory system. “Illusion of non-mediation” basically indicates that a person fails to perceive the existence of a medium and the user reacts as if the medium were not in his / her environment.

According to Coelho et al. [120] presence can be differentiated between a psychological experience (inner presence) and a technological experience (media presence). Media presence, also referred to as a rationalist point of view, “describes the sense of presence as a function of the experience of a given medium” ([120]; p. 27 and [121]). This means that presence is a “perceptual illusion of non-mediation produced by means of the disappearance of the medium from the conscious attention of the subject” ([120]; p. 28). Inner presence, also referred to as an ecological perspective, is a view which describes presence as a “neuropsychological phenomenon, evolved from the interplay of our biological and cultural inheritance, whose goal is the control of the human activity” ([120]; p. 28). According to Gorini et al. [122] *immersion* and *narrative* are important factors to create an effective experience with virtual reality. Narratives can create a meaning for the experiences of the participants, influence the way participants will assess them, change the person’s emotional condition, contribute to generate emotional responses and hence reinforce the person’s sense of inner presence, while immersion can increase illusion and mainly contributes to increased media presence [122]. Future studies should aim at analyzing in detail how narration and immersion are affected by the three technologies and apply a more elaborate conceptualization of presence. Measurement of presence could also be carried out in different ways. In order to measure presence, it might for instance be fruitful if external observers monitored the extent to which people fail to respond to real time occurrences while being immersed in the virtual environment (e.g. [121,123]).

More research could also be carried out on the circumstances under which the different technologies may influence attitude towards the game or towards the brands placed in the games. In the study on games by Kerrebroeck et al. [76], playing the HMD VR game led to higher vividness than playing the 2D version. Furthermore, Kerrebroeck et al. showed that there is a positive influence of vividness and presence as a mediator influencing the attitude toward the ad and the attitude toward the brand. The impact of vividness on attitude toward the advertisement and in further consequence on the attitude toward the ad and the mediating role of presence on the attitude toward the brands between the three technologies should be examined in a follow-up study [76].

This study used a student sample. Even though they clearly represent a possible target group of the video game used in our study, it would be interesting to see how other target groups (e.g. adolescents) react to the three technologies. Additionally, further research might want to focus on the influence of player variables (e.g. gender, prior game experience) in more detail. Another interesting aspect would be to analyze how different levels of prominence of the brand placements affect brand memory and attitudes in the different technologies. Given that players devote more cognitive resources to the game play in a VR video game, it could well be that a brand that is highly integrated in the game play (much more than in the game used in this study) could benefit from the enhanced technology. Additional research is clearly needed.

Future studies could also focus on how the player perceives the product type and design and the positioning and size of the brands in the game within the three conditions. Different product types and designs may influence the player in different ways (e.g. perception of the brand or brand attitude). Also, the size of the brand (e.g. small, bigger) and the positioning of the brands (e.g. in the corner, in the middle, at the top or bottom of the screen) could impact the player’s perception of the brands. Furthermore, it would be interesting to examine the different impact between the three technologies depending on product type variables, such as product type-game congruity or the involvement.

Future research should also be devoted to ergonomic or perceptual aspects of the games. In our video game, in all three conditions players steered the avatar, hence players experienced the action by guiding and observing the avatar, as opposed to a first-person perspective in which the gamer sees the action with the eyes of the avatar. Future studies may analyze the impact of the gamer perspective (especially a first-person game) when playing games in different technologies.

Another interesting field of research might be the analysis of video games that include additional sensory stimulation, e.g. scent, vibration or airflow. According to previous research, for instance, olfactory senses can have a strong influence on the selection, processing and consolidation of information and on memory (e.g. [124,125]). Finally, whereas experiences with 2D and 3D are widespread, the VR technology is less distributed and individuals do not have much experience with it. This might have influenced the results, since subjects in the VR condition might have experienced distraction due to the novelty of the technology.

## Supporting information

S1 Appendix Experimental design. (PDF)

S2 Appendix Questions and justification of items. (PDF)

S3 Appendix Supplementary figures and tables. (PDF)

S1 Data Data set of the main study. (SAV)

S2 Data Data set of the post hoc study. (SAV)

## References

[1] DiChristopher T. Digital gaming sales hit record $61 billion in 2015: Report [Internet]. Cnbc. 2016 [cited 2016 Jan 26]. Available from: http://www.cnbc.com/2016/01/26/digital-gaming-sales-hit-record-61-billion-in-2015-report.html

[2] BIU. Kauf: Umsatz digitale Spiele. Deutscher Gesamtmarkt für digitale Spiele im ersten Halbsjahr 2015 [Internet]. 2015 [cited 2016 May 5]. Available from: http://www.biu-online.de/de/fakten/marktzahlen-1-halbjahr-2015/kauf-umsatz-digitale-spiele.html.2015.

[3] Statista. Value of the global video games market from 2011 to 2020 (in billion U.S. dollars) [Internet]. 2018 [cited 2018 March 7]. Available from: https://www.statista.com/statistics/246888/value-of-the-global-video-game-market/

[4] Entertainment Software Association. Essential Facts: About the computer and video game industry [Internet]. Entertainment Software Association. 2016 [cited 2017 June 16]. Available from: http://essentialfacts.theesa.com/Essential-Facts-2016.pdf

[5] Casual Games Association. Southeast Asia Games Market. The World’s Fastest Growing Region. Casual Games Sector Report 2015. [Internet]. 2015 [cited 2016 May 22]. Available from: https://issuu.com/casualconnect/docs/southeastasia-report-2015

[6] Warman P. Rolling into the Southeast Asian Games Market. Sizing Opportunities in the World’s Fastest Growing Region [Internet]. 2015 [cited 2016 May 22]. Available from: https://newzoo.com/wp-content/uploads/2011/06/Newzoo_PG_Connects_Southeast_Asia_V1.pdf

[7] Simon J-P. Video games industry in China and cross cultural gaming [Internet]. 2015 [cited 2016 April 20]. Available from: http://www.inaglobal.fr/en/jeu-video/article/video-games-industry-china-and-cross-cultural-gaming-8446

[8] Statista. Genre breakdown of video game sales in the United States in 2016 [Internet]. 2016 [cited 2017 June 16]. Available from: https://www.statista.com/statistics/189592/breakdown-of-us-video-game-sales-2009-by-genre/

[9] Biocca F. Communication Within Virtual Reality: Creating a Space for Research. J Commun. 1992 12 1;42(4):5–22.

[10] Ijsselsteijn W, de Ridder H, Freeman J, Avons S, Bouwhuis D. Effects of Stereoscopic Presentation, Image Motion, and Screen Size on Subjective and Objective Corroborative Measures of Presence. Presence Teleoperators Virtual Environ. 2001;10(3):298–312. 10.1162/105474601300343621

[11] McMahan RP, Gorton D, Gresock J, McConnell W, Bowman DA. Separating the effects of level of immersion and 3D interaction techniques. Proc ACM Symp Virtual Real Softw Technol—VRST ‘06. 2006;108. doi:10.1145/1180495.1180518

[12] Rooney B, Hennessy E. Actually in the Cinema: A Field Study Comparing Real 3D and 2D Movie Patrons’ Attention, Emotion, and Film Satisfaction. Media Psychol. 2013;16:441–60. 10.1080/15213269.2013.838905

[13] Takatalo J, Kawai T, Kaistinen J, Nyman G, Häkkinen J. User Experience in 3D Stereoscopic Games. Media Psychol. 2011;14(4):387–414. 10.1080/15213269.2011.620538

[14] Yim MY-C, Cicchirillo VJ, Drumwright ME. The Impact of Stereoscopic Three-Dimensional (3-D) Advertising—The Role of Presence in Enhancing Advertising Effectiveness. J Advert. 2012;41(2):113–28. 10.2753/JOA0091-3367410208

[15] Wei W. Virtual Reality Enhanced Robotic Systems for Disability Rehabilitation. In: Hu F, Lu J., Zhang T, editors. Hershey, Pennsylvania: Idea Group Reference; 2016 p. 48–68.

[16] Osborne J. E3 2016 just deemed VR the future of gaming–whether you like it or not [Internet]. 2016 [cited 2016 August 11]. Available from: http://www.techradar.com/news/gaming/e3-2016-just-deemed-vr-the-future-of-gaming-whether-you-like-it-or-not-1323476

[17] Entertainment Software Assotiation. Essential Facts About the Computer and Video Game Industry [Internet]. 2017 [cited 2018 March 4]. Available from: http://www.theesa.com/wp-content/uploads/2017/09/EF2017

[18] Statista. Forecast augmented (AR) and virtual reality (VR) market size worldwide from 2016 to 2021 (in billion U.S. dollars) [Internet]. 2018 [cited 2018 March 4]. Available from: https://www.statista.com/statistics/591181/global-augmented-virtual-reality-market-size/

[19] Statista. Global VR gaming market size 2020 [Internet]. 2018 [cited 2018 March 4]. Available from: https://www.statista.com/statistics/499714/global-virtual-reality-gaming-sales-revenue/

[20] Statista. Virtual reality (VR) video gaming sales revenue worldwide from 2015 to 2020 (in billion U.S. dollars) [Internet]. 2018 [cited 2018 Feb 22]. Available from: https://www.statista.com/statistics/499714/global-virtual-reality-gaming-sales-revenue/

[21] Glass Z. The Effectiveness of Product Placement in Video Games. J Interact Advert. 2007;8(1):23–32. 10.1080/15252019.2007.10722134

[22] Williams KD. The effects of dissociation, game controllers, and 3D versus 2D on presence and enjoyment. Comput Human Behav. 2014;38:142–50. 10.1016/j.chb.2014.05.040

[23] Stephen AT, Coote LV. Brands in Action: The Role of Brand Placements in Building Consumer-Brand Identification In: American Marketing Association Proceedings. Chicago; 2005 p. 28.

[24] Cowley E, Barron C. When Product Placement Goes Wrong: The Effects of Program Liking and Placement Prominence. J Advert. 2008 4;37(1):89–98. 10.2753/JOA0091-3367370107

[25] Nelson MR. Recall of Brand Placements in Computer/Video Games. J Advert Res. 2002;42(2):80–92. 10.2501/JAR-42-2-80-92

[26] Terlutter R, Capella ML. The Gamification of Advertising: Analysis and Research Directions of In-Game Advertising, Advergames, and Advertising in Social Network Games. J Advert. 2013;42(2–3):95–112. 10.1080/00913367.2013.774610

[27] Balasubramanian SK, Karrh JA, Patwardhan H. Audience Response to Product Placements: An Integrative Framework and Future Research Agenda. J Advert. 2006 10;35(3):115–41. 10.2753/JOA0091-3367350308

[28] Planet Virtual Boy. Nintendo introduces video game players to three-dimensional worlds with new virtual reality video games [Internet]. 1994 [cited 2018 Feb 22]. Available from: http://www.planetvb.com/modules/advertising/?r17

[29] Stuart K. The 10 most influential handheld games consoles–in pictures [Internet]. 2017 [cited 2018 Mar 16]. Available from: https://www.theguardian.com/technology/gallery/2017/may/12/influential-handheld-games-consoles

[30] Short List. Everything you need to know about the Virtual Reality revolution [Internet]. [cited 2018 Feb 22]. Available from: https://www.shortlist.com/tech/gaming/everything-you-need-to-know-about-virtual-reality/6011

[31] Triberti S, Villani D, Riva G. Unconscious goal pursuit primes attitudes towards technology usage: A virtual reality experiment. Comput Human Behav. 2016 11;64:163–72. 10.1016/j.chb.2016.06.044

[32] Hollywood Branded Inc. Virtual Reality–The Future Of Product Placement? [Internet]. 2016 [cited 2018 March 4]. Available from: http://blog.hollywoodbranded.com/virtual-reality-the-future-of-product-placement

[33] VRS. Head-mounted Displays (HMDs) [Internet]. 2017 [cited 2018 March 4]. Available from: https://www.vrs.org.uk/virtual-reality-gear/head-mounted-displays/

[34] Digi-Capital. Augmented/Virtual Reality to hit $150 billion disrupting mobile by 2020 [Internet]. 2015 [cited 2016 May 8]. Available from: http://www.digi-capital.com/news/2015/04/augmentedvirtual-reality-to-hit-150-billion-disrupting-mobile-by-2020/#.VtQgEObDHE4

[35] Szoldra P. I’ve never felt emotions like this in a video game—until I tried VR [Internet]. 2016 [cited 2016 March 14]. Available from: http://www.techinsider.io/virtual-reality-is-2016-2

[36] Bradshaw T. Virtual reality: four ways it could change your world [Internet]. 2016 [cited 2016 March16]. Available from: http://www.ft.com/cms/s/0/0f7d7ecc-db47-11e5-a72f-1e7744c66818.html

[37] Google. Google Cardboard [Internet]. 2018 [cited 2018 Feb 23]. Available from: https://vr.google.com/cardboard/

[38] Microsoft. Microsoft HoloLens [Internet]. 2018 [cited 2018 Feb 22]. Available from: https://www.microsoft.com/en-us/hololens

[39] Collins K. Sony’s Project Morpheus is now officially called “PlayStation VR” [Internet]. 2015 [cited 2018 Feb 22]. Available from: http://www.wired.co.uk/article/sony-project-morpheus-now-playstation-vr

[40] Vive. Vive | Discover Virtual Reality Beyond Imagination [Internet]. 2018 [cited 2018 Feb 22]. Available from: https://www.vive.com/de/

[41] Huynh-Thu Q, Le Callet P, Barkowsky M. Video quality assessment: From 2D to 3D —Challenges and future trends. Image Process (ICIP), 2010 17th IEEE Int Conf Image Process. 2010;4025–8.

[42] Solimini AG. Are There Side Effects to Watching 3D Movies? A Prospective Crossover Observational Study on Visually Induced Motion Sickness. PLoS One. 2013;8(2). 10.1371/journal.pone.0056160 23418530 PMC3572028

[43] Harwell D. The Creepy, Inescapable Advertisments That Could Define Virtual Reality [Internet]. The Washington Post. 2016 [cited 2016 March 16]. Available from: http://gadgets.ndtv.com/wearables/features/the-creepy-inescapable-advertisements-that-could-define-virtual-reality-812463

[44] Nicas J. What Does Virtual Reality Do to Your Body and Mind? Wall Str J [Internet]. 2016 [cited 2016 April 22]; Available from: http://www.wsj.com/articles/what-does-virtual-reality-do-to-your-body-and-mind-1451858778

[45] Riva G, Waterworth J. Being Present in a Virtual World In: Grimshaw M, editor. The Oxford Handbook of Virtuality. Oxford University Press; 2014 p. 205–21. 10.1093/oxfordhb/9780199826162.013.015

[46] Riva G. Enacting interactivity: The role of presence. Emerging Communication: Studies in New Technologies and Practices in Communication. 2008 10: 97–114.

[47] Sanchez-Vives M V, Slater M. From presence to consciousness through virtual reality. Nat Rev Neurosci. 2005, 6:332–339. 10.1038/nrn1651 15803164

[48] Riva G, Waterworth JA, Waterworth EL, Mantovani F. From intention to action: The role of presence. New Ideas Psychol. 2011;29(1):24–37. 10.1016/j.newideapsych.2009.11.002

[49] Riva G, Botella C, Baños R, Mantovani F, García-Palacios A, Quero S, Serino S, Triberti S, Repetto C, Dakanalis A, Villani D, Gaggioli A. Presence-inducing media for mental health applications In: Lombard M, Biocca F, Freeman J, Ijsselsteijn W, Schaevitz R.J., editors. Immersed in Media: Telepresence Theory, Measurement and Technology. Springer International Publishing 2015 p. 283–332. 10.1007/978-3-319-10190-3_12

[50] Riva G, Mantovani F. From the body to the tools and back: a general framework for presence in mediated interactions. Interact Comput. 2012;24(4):203–10. 10.1016/j.intcom.2012.04.007

[51] Waterworth J, Riva G. Feeling present in the physical world and in computer-mediated environments. Springer; 2014 10.1057/9781137431677

[52] Triberti S, Riva G. Being Present in Action: A Theoretical Model About the “Interlocking” Between Intentions and Environmental Affordances. Front Psychol. 2016 1 22;6:2052 10.3389/fpsyg.2015.02052 26834670 PMC4722118

[53] Lombard M, Ditton T. At the Heart of It All: The Concept of Presence. J Comput Commun. 2006 6 23;3(2). 10.1111/j.1083-6101.1997.tb00072.x

[54] Nelson MR, Yaros RA, Keum H. Examining the Influence of Telepresence on Spectator and Player Processing of Real and Fictitious Brands in a Computer Game. J Advert. 2006 12 1;35(4):87–99. 10.2753/JOA0091-3367350406

[55] Kim T, Biocca F. Telepresence via Television: Two Dimensions of Telepresence May Have Different Connections to Memory and Persuasion. J Comput Commun. 1997 6 23;3(2). 10.1111/j.1083-6101.1997.tb00073.x

[56] Hernandez MD. A Model of Flow Experience as Determinant of Positive Attitudes Toward Online Advergames. J Promot Manag. 2011;17:315–26. 10.1080/10496491.2011.596761

[57] Bolls PD, Lang A, Potter RF. The Effects of Message Valence and Listener Arousal on Attention, Memory, and Facial Muscular Responses to Radio Advertisements. Communic Res. 2001 10 1;28(5):627–51. 10.1177/009365001028005003

[58] Limperos A, Waddell TF, Ivory AH, Ivory JD. Psychological and Physiological Responses to Stereoscopic 3D Presentation in Handheld Digital Gaming: Comparing the Experiences of Frequent and Infrequent Game Players. Presence Teleoperators Virtual Environ. 2014 11 1;23(4):341–53. 10.1162/PRES_a_00204

[59] Meehan M, Insko B, Whitton M, Brooks Jr FP. Physiological measures of presence in stressful virtual environments. Vol. 21, ACM Transactions on Graphics; Proceedings of ACM SIGGRAPH 2002, July 23, 2002—July 26, 2002. 2002. p. 645–52. doi: 10.1145/566654.566630

[60] Phillips L, Interrante V, Kaeding M, Ries B, Anderson L. Correlations Between Physiological Response, Gait, Personality, and Presence in Immersive Virtual Environments. Presence Teleoperators Virtual Environ. 2012;21(2):119–41. 10.1162/PRES_a_00100

[61] Slater M, Antley A, Davison A, Swapp D, Guger C, Barker C, et al A Virtual Reprise of the Stanley Milgram Obedience Experiments. Rustichini A, editor. PLoS One. 2006 12 20;1(1):e39 10.1371/journal.pone.0000039 17183667 PMC1762398

[62] Ferdig RERE, Klinger K, Roth KM, Boyer M, Appicello A, Environment V, et al Handbook of Research on Effective Electronic Gaming in Education. New York: IGI Global; 2008.

[63] Persky S, McBride CM. Immersive Virtual Environment Technology: A Promising Tool for Future Social and Behavioral Genomics Research and Practice. Health Commun. 2009 12;24(8):677–82. 10.1080/10410230903263982 20183376 PMC2829714

[64] Schild J, LaViola JJ Jr., Masuch M. Understanding user experience in stereoscopic 3D games. Conf Hum Factors Comput Syst—Proc. 2012;89–98. 10.1145/2207676.2207690

[65] Persky S, Blascovich J. Immersive Virtual Video Game Play and Presence: Influences on Aggressive Feelings and Behavior. Presence Teleoperators Virtual Environ. 2008;17(1):57–72. 10.1162/pres.17.1.57

[66] Solomon MR, Bamossy G, Askegaard S, Hogg MK. Consumer Behaviour: A European Perspective. New Jersey: Prentice Hall International 2007.

[67] Lewis B, Porter L. In-Game Advertising Effects. J Interact Advert. 2010 3 1;10(2):46–60. 10.1080/15252019.2010.10722169

[68] Waiguny MKJ, Nelson MR, Marko B. How Advergame Content Influences Explicit and Implicit Brand Attitudes: When Violence Spills Over. J Advert. 2013;42(2–3):155–69. 10.1080/00913367.2013.774590

[69] van Reijmersdal E a., Rozendaal E, Buijzen M. Effects of Prominence, Involvement, and Persuasion Knowledge on Children’s Cognitive and Affective Responses to Advergames. J Interact Mark. 2012 2;26(1):33–42. 10.1016/j.intmar.2011.04.005

[70] Wise K, Bolls PD, Kim H, Venkataraman A, Meyer R. Enjoyment of Advergames and Brand Attitudes: the Impact of Thematic Relevance. J Interact Advert. 2008;9(1):27–36. 10.1080/15252019.2008.10722145

[71] Dens N, Pelsmacker P De, Wouters M, Purnawirawan N. Do you like What you Recognize? J Advert Advert. 2012;41(3):35–53. 10.2753/JOA0091-3367410303

[72] Li H, Daugherty T, Biocca F. Impact of 3-D advertising on product knowledge, brand attitude, and purchase intention: The mediation role of presence. J Advert. 2002;31(3):43–57.

[73] Choi YK, Taylor CR. How do 3-dimensional images promote products on the Internet? J Bus Res. 2014;67(10):2164–70.

[74] Debbabi S, Daassi M, Baile S. Effect of online 3D advertising on consumer responses: the mediating role of telepresence. J Mark Manag. 2010;26(9–10):967–92.

[75] Lee K-Y, Li H, Edwards SM. The Effect of 3-D Product Visualisation on the Strength of Brand Attitude. Int J Advert. 2012;31(3):377–96.

[76] Van Kerrebroeck H, Brengman M, Willems K. When brands come to life: experimental research on the vividness effect of Virtual Reality in transformational marketing communications. Virtual Real. 2017;21(4):177–91.

[77] Gupta PB, Lord KR. Product Placement in Movies: The Effect of Prominence and Mode on Audience Recall. J Curr Issues Res Advert. 1998;20(1):47–59. 10.1080/10641734.1998.10505076

[78] D’Astous A, Chartier F. A Study of Factors Affecting Consumer Evaluations and Memory of Product Placements in Movies. J Curr Issues Res Advert. 2000 9;22(2):31–40. 10.1080/10641734.2000.10505106

[79] Law S, Braun KA. I’ll have what she’s having: Gauging the impact of product placements on viewers. Psychol Mark. 2000;17(12), 1059–75. 10.1002/1520-6793

[80] Walsh P, Kim Y, Ross SD. Brand Recall and Recognition: A Comparison of Television and Sport Video Games as Presentation Modes. Sport Mark Q [Internet]. 2008;17(4):201–8.

[81] Aaker DA. Building strong brands. New York: Simon and Schuster; 2012.

[82] Lang A. The limited capacity model of mediated message processing. J Commun. 2000 3 1;50(1):46–70. 10.1093/joc/50.1.46

[83] Grigorovici DM, Constantin CD. Experiencing Interactive Advertising beyond Rich Media: Impacts of Ad Type and Presence on Brand Effectiveness in 3D Gaming Immersive Virtual Environments. J Interact Advert. 2004;4(3):N.PAG. 10.1080/15252019.2004.10722091

[84] Lee M, Faber RJ. Effects of Product Placement in On-line Games on Brand Memory: A Perspective of the Limited-Capacity Model of Attention. J Advert. 2007;36(4):75–90. 10.2753/JOA0091-3367360406

[85] Herrewijn L, Poels K. The impact of social setting on the recall and recognition of in-game advertising. Comput Hum Behav J. 2014;53:544–55. 10.1016/j.chb.2014.06.012

[86] Mun S, Park M-C, Park S, Whang M. SSVEP and ERP measurement of cognitive fatigue caused by stereoscopic 3D. Neurosci Lett. 2012;525(2):89–94. 10.1016/j.neulet.2012.07.049 22884933

[87] Yim MY-C, Abdourazakou Y, Sauer PL, Park S-Y. Modelling the dimensionality effects on brand placement effectiveness in stereoscopic 3-D versus 2-D sports games. Int J Advert. 2017; 12:1–26. 10.1080/02650487.2017.1347366

[88] Sebrechts MM, Cugini J V, Laskowski SJ, Vasilakis J, Miller MS. Visualization of search results: a comparative evaluation of text, 2D, and 3D interfaces. In: Hearst M, Gey FC, Tong R, editors. Proceedings of the 22nd annual international ACM SIGIR conference on Research and development in information retrieval. New York, NY: ACM; 1999. p. 3–10.

[89] Rajae-Joordens RJE. Measuring experiences in gaming and TV applications In: Westerink J, Ouwerkerk M, Overbeek TJM, Pasveer WF, editors. Probing Experience. Dordrecht, The Netherlands: Springer; 2008 p. 77–90.

[90] van Der Land S, Schouten AP, Feldberg F, Van Den Hooff B, Huysman M. Lost in space? Cognitive fit and cognitive load in 3D virtual environments. Comput Human Behav. 2013;29(3):1054–64. 10.1016/j.chb.2012.09.006

[91] Terlutter R, Diehl S, Koinig I, Waiguny MKJ. Positive or Negative Effects of Technology Enhancement for Brand Placements? Memory of Brand Placements in 2D, 3D, and 4D Movies. Media Psychol. 2016;19(4):505–33. 10.1080/15213269.2016.1142377

[92] Oxford N. What’s the Definition of An Action Game? [Internet]. 2017 [cited 2018 March 23]. Available from: https://www.lifewire.com/nintendo-action-game-1126179

[93] Fritz CO, Morris PE, Richler JJ. Effect size estimates: current use, calculations, and interpretation. J Exp Psychol Gen. 2012;141(1):2 10.1037/a0024338 21823805

[94] Richardson JTE. Eta squared and partial eta squared as measures of effect size in educational research. Educ Res Rev. 2011;6(2):135–47.

[95] Yatani K. Effect Sizes and Power Analysis in HCI In: Robertson J, Kaptein M, editors. Modern Statistical Methods for HCI. Springer International Publishing Switzerland; 2016 p. 87–110.

[96] Lakens D. Calculating and reporting effect sizes to facilitate cumulative science: A practical primer for t-tests and ANOVAs. Front Psychol. 2013 11 26;4:863 10.3389/fpsyg.2013.00863 24324449 PMC3840331

[97] Hedges LV. Distribution theory for Glass’s estimator of effect size and related estimators. J Educ Stat. 1981;6(2):107–128.

[98] Hedges LV. Estimation of effect size from a series of independent experiments. Psychol Bull. 1982;92(2):490–499.

[99] Rosnow RL, Rosenthal R. Effect sizes: Why, when, and how to use them. Zeitschrift für Psychol Psychol. 2009;217(1):6–14.

[100] Cohen J. Statistical power analysis for the behavioral sciences. Erlbaum Associates, Hillsdale; 1988.

[101] Murphy KR, Myors B, Wolach A. Statistical power analysis: A simple and general model for traditional and modern hypothesis tests. Routledge; 2014.

[102] Entertainment Software Association. Essential Facts About the Computer and Video Game Industry [Internet]. Entertainment Software Association. 2015 [cited 2016 May 7]. Available from: http://www.theesa.com/wp-content/uploads/2015/04/ESA-Essential-Facts-2015.pdf

[103] Johnson D, Gardner J, Sweetser P. Motivations for videogame play: predictors of time spent playing. Comput Human Behav. 2016;63:805–12.

[104] Gross ML. Advergames and the effects of game-product congruity. Comput Human Behav. 2010;26(6):1259–65.

[105] Shamdasani PN, Stanaland AJS, Tan J. Location, location, location: Insights for advertising placement on the web. J Advert Res. 2001;41(4):7–21.

[106] Rheinberg F, Vollmeyer R, Engeser S. Die Erfassung des Flow-Erlebens In: Stiensmeiser-Pelster J, Rheinberg F, editors. Diagnostik von Motiv und Selstkonzept. Göttingen: Hogrefe; 2003 p. 261–279.

[107] Grier JB. Nonparametric indexes for sensitivity and bias: computing formulas. Psychol Bull. 1971;75(6):424–9. 5580548 10.1037/h0031246

[108] Obermiller C, Spangenberg ER. Development of a scale to measure consumer skepticism toward advertising. J Consum Psychol. 1998;7(2):159–86.

[109] Porter CE, Donthu N. Using the technology acceptance model to explain how attitudes determine Internet usage: The role of perceived access barriers and demographics. J Bus Res. 2006 9;59(9):999–1007. 10.1016/j.jbusres.2006.06.003

[110] Chandler P, Sweller J. Cognitive load theory and the format of instruction. Cogn Instr. 1991;8(4):293–332.

[111] Gibson B. Can Evaluative Conditioning Change Attitudes toward Mature Brands? New Evidence from the Implicit Association Test. J Consum Res. 2008;35(1):178–88.

[112] Jones T, Moore T, Choo J. The impact of virtual reality on chronic pain. PLoS One. 2016;11(12):1–10. 10.1371/journal.pone.0167523 PMC5172565 27997539

[113] Merhi O, Faugloire E, Flanagan M, Stoffregen TA. Motion Sickness, Console Video Games, and Head-Mounted Displays. Hum Factors J Hum Factors Ergon Soc. 2007;49(5):920–34. 10.1518/001872007X230262 17915607

[114] Paas FGWC, Van Merriënboer JJG. The efficiency of instructional conditions: An approach to combine mental effort and performance measures. Hum Factors. 1993;35(4):737–43. 10.1177/001872089303500412

[115] Brünken R, Plass JL, Leutner D. Direct measurement of cognitive load in multimedia learning. Educ Psychol. 2003;38(1):53–61. 10.1207/S15326985EP3801_7 12053529

[116] Winkler T, Buckner K. Receptiveness of Gamers to Embedded Brand Messages in Advergames: Attitudes towards Product Placement. J Interact Advert. 2006;7(1):37–46. 10.1080/15252019.2006.10722123

[117] Choi YK, Lee SM, Li H. Audio and Visual Distractions and Implicit Brand Memory: A Study of Video Game Players. J Advert. 2013;42(2–3):219–27. 10.1080/00913367.2013.775798

[118] Dardis FE, Schmierbach M, Limperos AM. The Impact of Game Customization and Control Mechanisms on Recall of Integral and Peripheral Brand Placements in Videogames. J Interact Advert. 2012;12(2):1–12. 10.1080/15252019.2012.10722192

[119] Zahorik P, Jenison RL. Presence as Being-in-the-World. Presence Teleoperators Virtual Environ. 1998;7(1):78–89. 10.1162/105474698565541

[120] Coelho C, Tichon JG, Hine TJ, Wallis GM, Riva G. Media presence and inner presence: The Sense of presence in virtual reality technologies In: Riva G, Anguera MT, Wiederhold BK, Mantovani F, editors. From Communication to Presence: Cognition, Emotions and Culture towards the Ultimate Communicative Experience Festschrift in honor of Luigi Anolli. IOS Press, Amsterdam; 2006 p. 25–45.

[121] Slater M, Usoh M. Presence in immersive virtual environments. Proc IEEE Virtual Real Annu Int Symp. 1993;(c):90–6.

[122] Gorini A, Capideville CS, De Leo G, Mantovani F, Riva G. The Role of Immersion and Narrative in Mediated Presence: The Virtual Hospital Experience. Cyberpsychology, Behav Soc Netw. 2010 7 22;14(3):99–105. 10.1089/cyber.2010.0100 20649451

[123] Held RM, Durlach NI. Telepresence. Presence Teleoperators Virtual Environ. 1992 1 1;1(1):109–12.

[124] Cahill L, Babinsky R, Markowitsch HJ, McGaugh JL. The amygdala and emotional memory. Nature. 1995 9 28;377(6547):295–6. 10.1038/377295a0 7566084

[125] Eichenbaum H. Olfactory perception and memory In: Llinás RR, Churchland PS, editors. The mind-brain continuum: sensory processes. Cambridge, MA: MIT Press; 1996 p. 173–202.

