# Supplementary material for: The same video game in 2D, 3D or virtual reality – How does technology impact game evaluation and brand placements?
Source: PLoS One. 2018 Jul 20;13(7):e0200724. doi: 10.1371/journal.pone.0200724 (PMC6054385; doi:10.1371/journal.pone.0200724)

S1 Appendix. Experimental design.

Before conducting the main study, a first pre-test was carried out to develop and to test the video game in all three conditions (n=10 students). 237 students from a midsize university in Europe participated in the main study, held in computer labs on the campus. Participants were randomly assigned to one of the three game conditions (2D, 3D, VR). Afterwards the subjects filled out the same electronic questionnaire in another room. A post hoc study (n=53) was carried out in order to gain more insights into the cognitive load and some physical reactions (dizziness and motion-sickness) while playing the video game. In addition, the aim was to confirm the memory results of the main study.

Figure A. The experimental design.

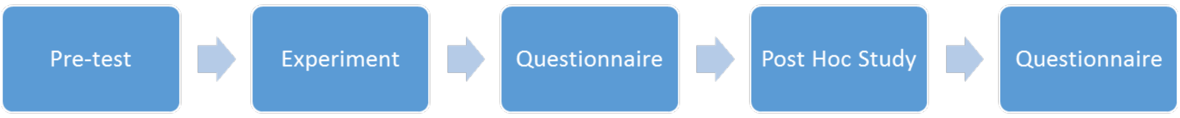

Figure B. A VR-participant in the computer lab.

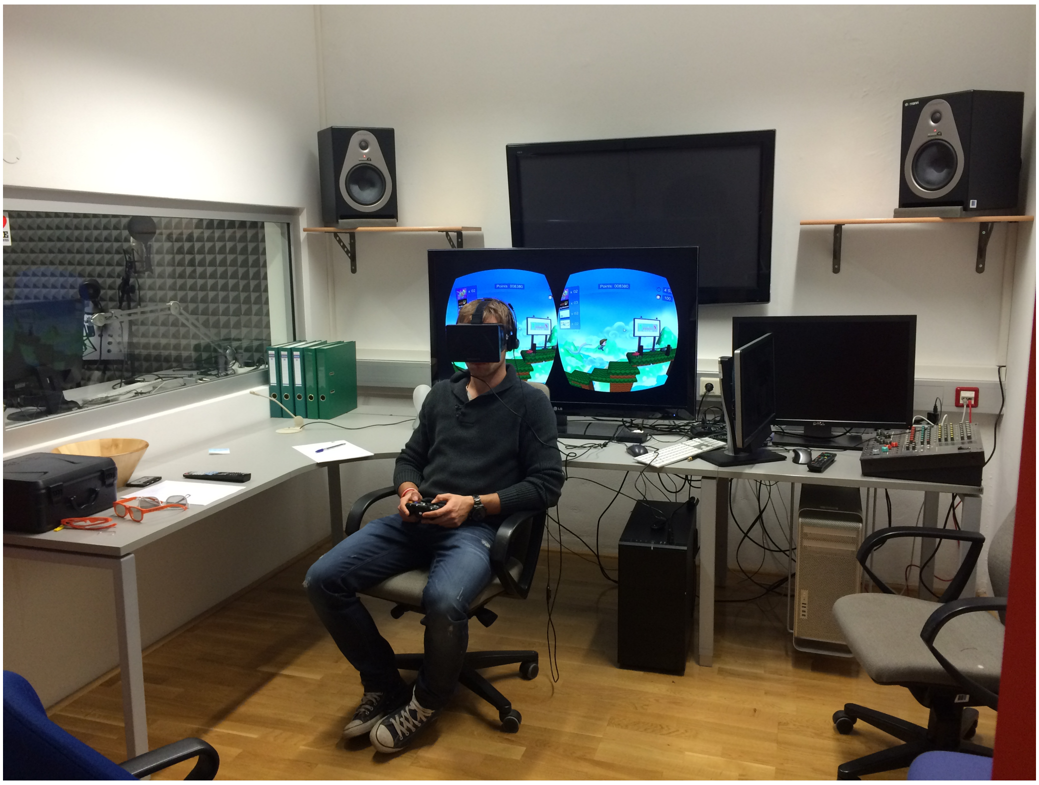

Supplement: S1 Appendix — (PDF) [file pone.0200724.s001.pdf]
