# Supplementary material for: The same video game in 2D, 3D or virtual reality – How does technology impact game evaluation and brand placements?
Source: PLoS One. 2018 Jul 20;13(7):e0200724. doi: 10.1371/journal.pone.0200724 (PMC6054385; doi:10.1371/journal.pone.0200724)
Supplement: S2 Appendix — (PDF) [file pone.0200724.s002.pdf]

S2 Appendix. Questions and justification of items.

(Original language and translation for the paper.)

EINSTELLUNG GEGENÜBER DEM SPIEL / ATTITUDE TOWARD THE GAME Gross, 2010)

[F1] Die folgenden Fragen beziehen sich auf das Video Game, das Sie soeben gespielt haben. Bitte beurteilen Sie das Spiel anhand der vorgegebenen Eigenschaftswörter, z.B. von -3 (nicht ansprechend) bis +3 (ansprechend).

Wie hat Ihnen das Spiel gefallen? Das Spiel war…

		-3	-2	-1	0	+1	+2	+3	
F1_1	nicht ansprechend	o	o	o	o	o	o	o	ansprechend
F1_2	unangenehm	o	o	o	o	o	o	o	angenehm
F1_3	langweilig	o	o	o	o	o	o	o	dynamisch
F1_4	nicht anziehend	o	o	o	o	o	o	o	anziehend
F1_5	nicht unterhaltsam	o	o	o	o	o	o	o	unterhaltsam
F1_6	bedrückend	o	o	o	o	o	o	o	erquickend

[F1] The following questions refer to the video game, which you have just played. Please, evaluate the game on basis of the given adjectives, e.g. from -3 (unappealing) to +3 (appealing).

How did you like the game? The game was…

		-3	-2	-1	0	+1	+2	+3	
F1_1	unappealing	o	o	o	o	o	o	o	appealing
F1_2	unpleasant	o	o	o	o	o	o	o	pleasant
F1_3	dull	o	o	o	o	o	o	o	dynamic
F1_4	unattractive	o	o	o	o	o	o	o	attractive
F1_5	not enjoyable	o	o	o	o	o	o	o	enjoyable
F1_6	depressing	o	o	o	o	o	o	o	refreshing

ERREGUNG / AROUSAL (Hernandez et al., 2011)

[F2] Bitte kreuzen Sie anhand der vorgegebenen Eigenschaftswörter an, inwieweit die folgenden Statements auf Sie zutreffen.

Während ich das Video Game spielte, fühlte ich mich…

		-3	-2	-1	0	+1	+2	+3	
F2_1	aufgeregt	o	o	o	o	o	o	o	gelassen
F2_2	angeregt	o	o	o	o	o	o	o	entspannt
F2_3	hellwach	o	o	o	o	o	o	o	ruhig

[F2] Please, choose the adjective, which is true for you.

While I played the game I felt…

		-3	-2	-1	0	+1	+2	+3	
F2_1	excited	o	o	o	o	o	o	o	calm
F2_2	stimulated	o	o	o	o	o	o	o	relaxed
F2_3	alert	o	o	o	o	o	o	o	soothed

RECALL (Nelson et al., 2006)

[F4] An welche Marken und Produkte, die im Spiel erschienen sind, können Sie sich erinnern? Schreiben Sie die Produkte- oder Markennamen oder Kategorien auf.

____

[F4] Which brands or products can you remember? Write down the brand or product names or categories.

____

RECOGNITION (Nelson et al., 2006)

[F5-F12] Welche Logo haben Sie während des Spiels gesehen? Entscheiden Sie sich bitte für ein Logo, das Sie Ihrer Meinung nach gesehen haben.

[F5-F12] Which logo have you seen while you have played the game? Choose the one, which you think you have seen.

[F5]

Airline	
o Air Europe	AirEuropa
o Miracle Air	Miracle Air
o Copa Airlines	Copa Airlines
o Sonstiges / Others / Keine Antwort / No answer	

[F6]

Chocolate	
o Sir Choconut	Sir Choconut
o Oh Henry!	Oh HENRY!
o Reese's Crispy Crunchy Bar	New! Reese's Crispy Crunchy Bar
o Sonstiges / Others / Keine Antwort / No answer	

[F7]

Bank	
o Bank of the West	BANK OF THE WEST BNP PARIBAS GROUP
o Companion Bank	Companion Bank
o Swedbank	Swedbank
o Sonstiges / Others / Keine Antwort / No answer	

[F8]

Energy Drink	
o Rockstar	
o Power Up	
o XL Power	
o Sonstiges / Others / Keine Antwort / No answer	

[F9]

Coffee	
o Café del Colombia	
o Delicious Coffee	
o Royal Coffee	
o Sonstiges / Others / Keine Antwort / No answer	

[F10]

Fitness Center	
o Training & Fitness Club	
o Cabo Fitness Club	
o Fitness Forever	
o Sonstiges / Others / Keine Antwort / No answer	

[F11]

Nachos	
o Crispy Nachos	
o Tacos Y Nachos	
o Nacho Nachos	
o Sonstiges / Others / Keine Antwort / No answer	

[F12]

Smartphone	
o OnePlus	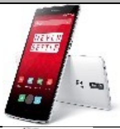
o ZTE	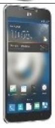
o QT1	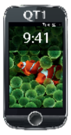
o Sonstiges / Others / Keine Antwort / No answer	

EINSTELLUNG GEGENÜBER DEM PRODUKT / ATTITUDE TOWARD THE PRODUCT, α=.xxx (in Anlehnung an / adapted from Shamdasani et al., 2001)

[F13, F16, F19, F22] Bitte sehen Sie sich das Logo von "Crispy Nachos"/ „Sir Choconut" / „QT1" / „XL Power", welches auch im Spiel vorgekommen ist, an und lesen Sie sich anschließend die untenstehenden Aussagen genau durch. Stufen Sie bitte anhand der Skala ab, inwieweit Sie den folgenden Aussagen zustimmen.

		-3	-2	-1	0	+1	+2	+3	
F13/F16/F19/F22_1	Das Produkt ist schlecht	o	o	o	o	o	o	o	gut
F13/F16/F19/F22_2	Meine Gefühle gegenüber dem Produkt sind negativ	o	o	o	o	o	o	o	positiv
F13/F16/F19/F22_3	Das Produkt ist schrecklich	o	o	o	o	o	o	o	schön
F13/F16/F19/F22_4	Das Produkt ist unsympathisch	o	o	o	o	o	o	o	sympathisch
F13/F16/F19/F22_5	Das Produkt ist nicht ansprechend	o	o	o	o	o	o	o	ansprechend
F13/F16/F19/F22_6	Ich bin mit dem Produkt nicht einverstanden	o	o	o	o	o	o	o	einverstanden

[F13, F16, F19, F22] Please look at the logo of "Crispy Nachos"/ „Sir Choconut" / „QT1" / „XL Power" precisely and read through the statements below. Please, mark all answers that apply.

		-3	-2	-1	0	+1	+2	+3	
F13/F16/F19/F22_1	This is a bad product	o	o	o	o	o	o	o	This is a good product
F13/F16/F19/F22_2	I feel negative toward the product	o	o	o	o	o	o	o	I feel positive toward the product
F13/F16/F19/F22_3	The product is awful	o	o	o	o	o	o	o	The product is nice
F13/F16/F19/F22_4	The product is unpleasant	o	o	o	o	o	o	o	The product is pleasant
F13/F16/F19/F22_5	The product is unattractive	o	o	o	o	o	o	o	The product is attractive
F13/F16/F19/F22_6	I disapprove of the product	o	o	o	o	o	o	o	I approve of the product

PRÄSENZ / PRESENCE (Rheinberg et al., 2013)

[F25] Bitte beantworten Sie, wie sehr die folgende Aussage auf Sie zutrifft, indem Sie die entsprechende Zahl von 1 (trifft gar nicht zu) bis 7 (trifft völlig zu) ankreuzen. Beziehen Sie sich bei der Beantwortung der Frage bitte auf die soeben durchgeführte Tätigkeit, das Spielen des Video Games.

		trifft gar nicht zu						trifft völlig zu	keine Antwort
F25	Ich war ganz vertieft in das, was ich gerade machte.	1	2	3	4	5	6	7	8

[F25] Please, mark the answer that applies, from 1 (strongly disagree) to 7 (strongly agree). When answering the question, please refer to the just performed task, playing the video game.

		strongly disagree						strongly agree	no answer
F25	I was totally absorbed in what I was doing.	1	2	3	4	5	6	7	8

SKEPSIS GEGENÜBER WERBUNG / SCEPTICISM TOWARD ADVERTISING (Obermiller & Spangenberg, 1998)

[F29] Bitte kreuzen Sie an, inwiefern die folgenden Statements auf Sie zutreffen, von 1 (trifft gar nicht zu) bis 7 (trifft völlig zu).

		trifft gar nicht zu						trifft völlig zu	keine Antwort
F29_1	Wir können uns darauf verlassen, die Wahrheit in den meisten Werbungen gesagt zu bekommen.	1	2	3	4	5	6	7	8
F29_2	Ziel der Werbung ist es, den Konsumenten zu informieren.	1	2	3	4	5	6	7	8
F29_3	Ich glaube, Werbung ist informativ.	1	2	3	4	5	6	7	8
F29_4	Werbung ist im Allgemeinen wahrheitsgetreu.	1	2	3	4	5	6	7	8
F29_5	Werbung ist eine verlässliche Informationsquelle für Qualität und Leistungen eines Produktes.	1	2	3	4	5	6	7	8
F29_6	Werbung ist die Wahrheit, gut erzählt.	1	2	3	4	5	6	7	8
F29_7	Im Allgemeinen zeigt Werbung ein wahres Bild vom beworbenen Produkt.	1	2	3	4	5	6	7	8
F29_8	Nachdem ich Werbung gesehen habe, habe ich das Gefühl, sorgfältig informiert zu sein.	1	2	3	4	5	6	7	8
F29_9	Die meisten Werbungen bieten Konsumenten wesentliche Informationen.	1	2	3	4	5	6	7	8

[F29] Please, mark all answers that apply, from 1 (strongly disagree) to 7 (strongly agree).

		strongly disagree						strongly agree	no answer
F29_1	We can depend on getting the truth in most advertising.	1	2	3	4	5	6	7	8
F29_2	Advertising's aim is to inform the	1	2	3	4	5	6	7	8

	consumer.								
F29_3	I believe advertising is informative.	1	2	3	4	5	6	7	8
F29_4	Advertising is generally truthful.	1	2	3	4	5	6	7	8
F29_5	Advertising is a reliable source of information about the quality and performance of products.	1	2	3	4	5	6	7	8
F29_6	Advertising is truth well told.	1	2	3	4	5	6	7	8
F29_7	In general, advertising presents a true picture of the product being advertised.	1	2	3	4	5	6	7	8
F29_8	I feel I've been accurately informed after viewing most advertisements.	1	2	3	4	5	6	7	8
F29_9	Most advertising provides consumers with essential information.	1	2	3	4	5	6	7	8

ALLGEMEINE EINSTELLUNG GEGENÜBER VIDEOSPIELEN / GENERAL ATTITUDE TOWARD VIDEO GAMES (in Anlehnung an/ adapted from Porter & Donthu, 2006)

[F32] Welche Gefühle haben Sie gegenüber Videospielen im Allgemeinen?

sehr negative						sehr positive	keine Antwort
1	2	3	4	5	6	7	8

[F32] In general, what kind of feelings do you have toward video games?

very negative						very positive	no answer
1	2	3	4	5	6	7	8

VIDEO GAME LITERACY (edited by the researcher)

[F33] Wie würden Sie sich im Umgang mit Videospielen im Allgemeinen selbst einstufen?

überhaupt keine Kenntnisse						sehr gute Kenntnisse	keine Antwort
1	2	3	4	5	6	7	8

[F33] How much skills do you have by using video games?

no skills at all						very good skills	no answer
1	2	3	4	5	6	7	8

NUTZUNGSVERHALTEN / USAGE BEHAVIOR (edited by the researcher)

[F34] Wenn Sie an Ihre Spielehäufigkeit denken, wie viele Stunden spielen die durchschnittlich Videospiele?

Durchschnittliche Spielhäufigkeit **in Stunden pro Monat __________**

[F34] If you think about your video gaming frequency, how many hours do you play video games on average?

Average playing frequency **in hours per month __________**

[F35] Wie viel Erfahrung mit Spielen dieser Art (jump'n'run Spiele), wie Sie es soeben gespielt haben, haben Sie?

überhaupt keine Erfahrung						sehr viel Erfahrung	keine Antwort
1	2	3	4	5	6	7	8

[F35] How much experience with games of this type (jump'n'run games), as you have just played, do you have?

no experience at all						very much experience	no answer
1	2	3	4	5	6	7	8

DEMOGRAPHISCHE ANGABEN / SOCIODEMOGRAPHIC DATA (edited by the researcher)

[F36] Geschlecht

o weiblich

o männlich

[F36] Gender

o female

o male

[F37] Wie alt sind Sie? Bitte geben Sie Ihr Geburtsjahr an: _____

[F37] How old are you? Year of birth: _____
