# Supplementary material for: The same video game in 2D, 3D or virtual reality – How does technology impact game evaluation and brand placements?
Source: PLoS One. 2018 Jul 20;13(7):e0200724. doi: 10.1371/journal.pone.0200724 (PMC6054385; doi:10.1371/journal.pone.0200724)

S3 Appendix. Supplementary figures and tables.

Table S1. ANOVAs comparing the three technology groups (2D, 3D, VR)		
**Item**	**F**	**p**
Presence in the 2D, 3D and VR video game	5.104	<0.01
Attitude towards the video game	.365	ns
Arousal while playing the video game	.984	ns
Attitude towards the brands placed in the video game_nachos	.437	ns
Attitude towards the brands placed in the video game_chocolate	1.340	ns
Attitude towards the brands placed in the video game_smartphone	1.186	ns
Attitude towards the brands placed in the video game_energy drink	.748	ns
Memory_recall	8.514	<0.01
Memory_recognition	14.571	<0.01

Figure S1. Presence in the 2D, 3D and VR video game (F=5.104, p<0.01)

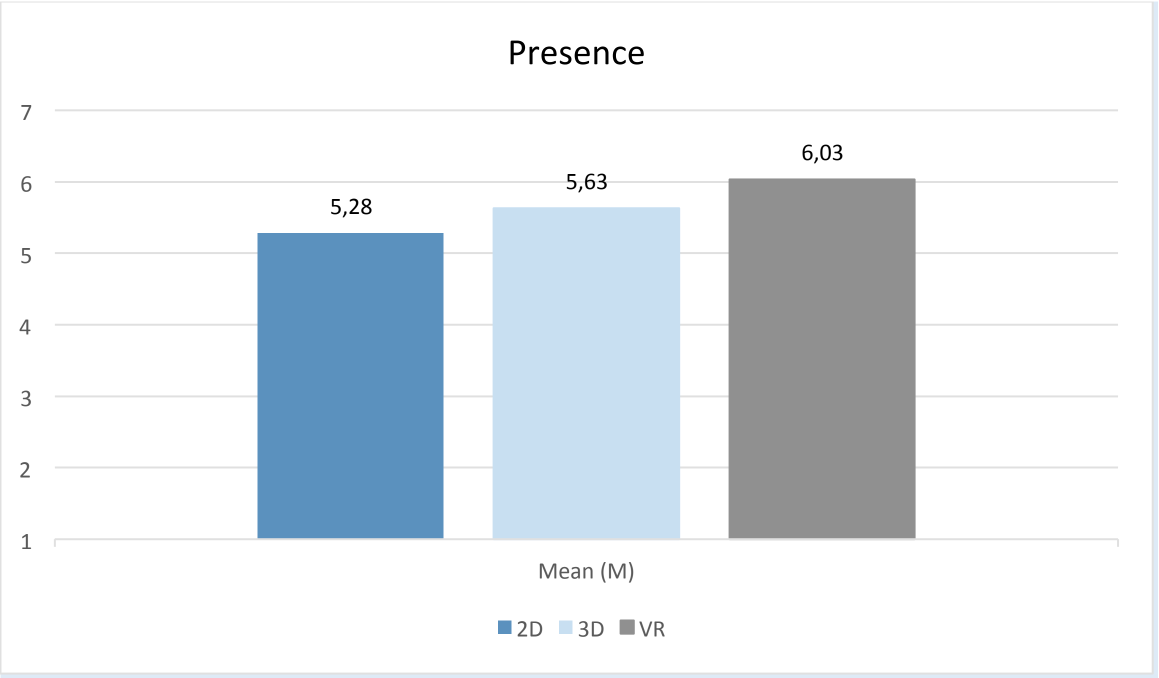

Figure S2. Arousal while playing the video game (F=.984, ns)

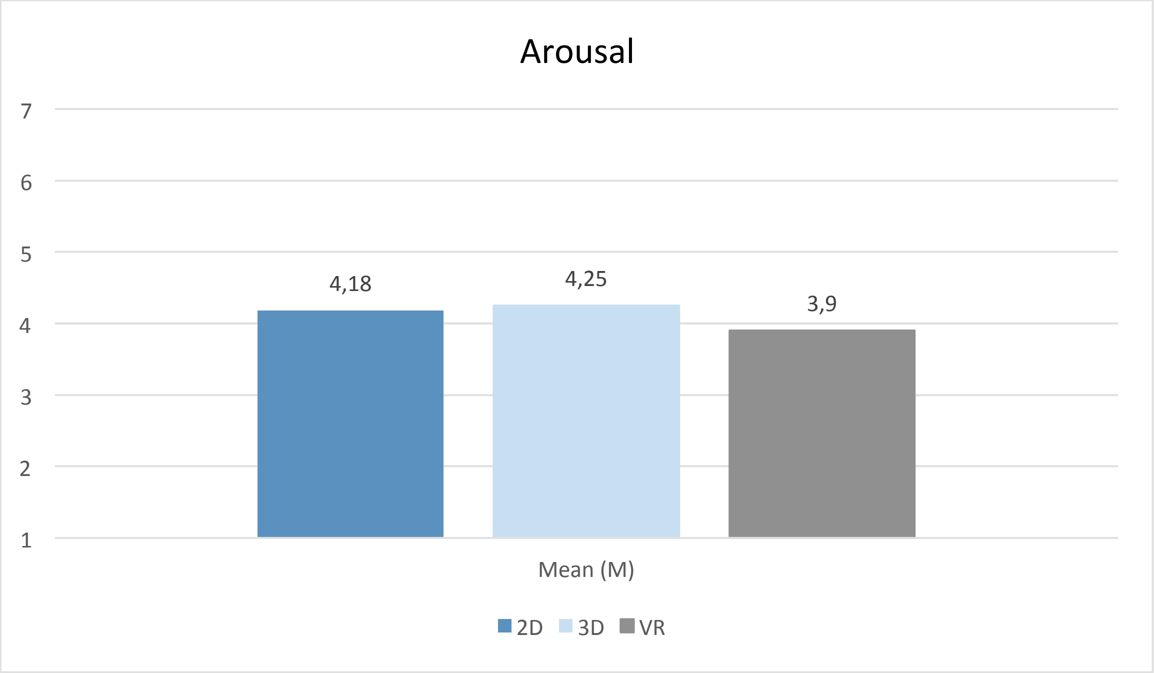

Figure S3. Attitude towards the video game (F=.365, ns)

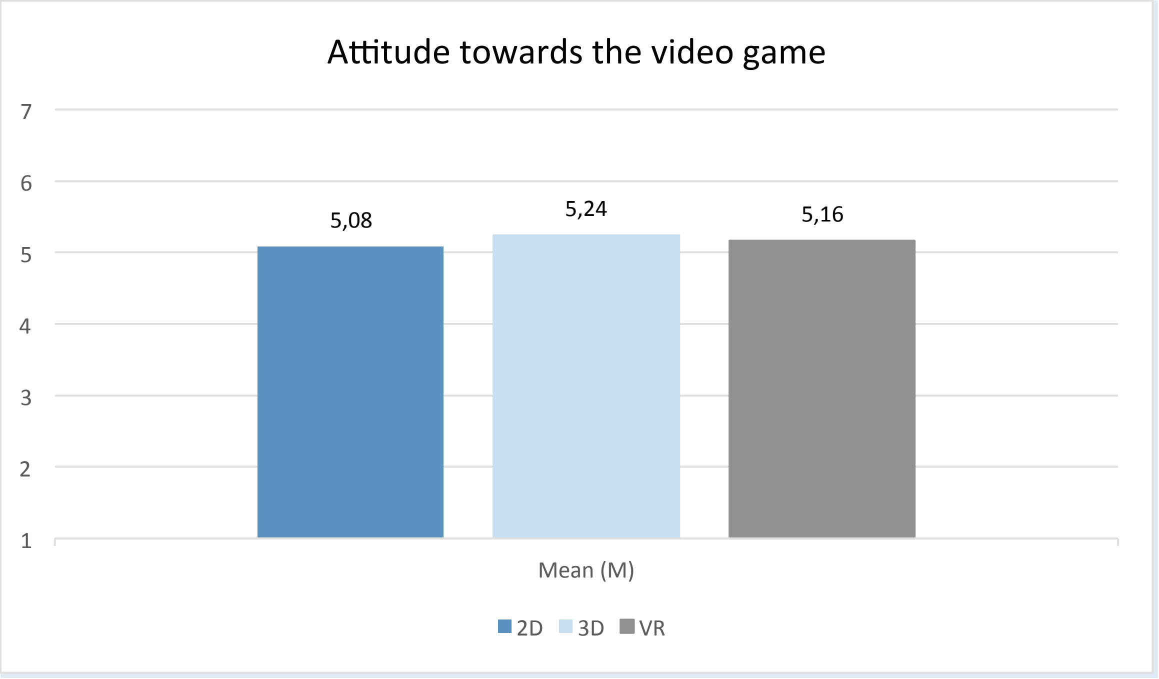

Figure S4. Memory for the placed brands (Recall: F=8.514, p<.01)

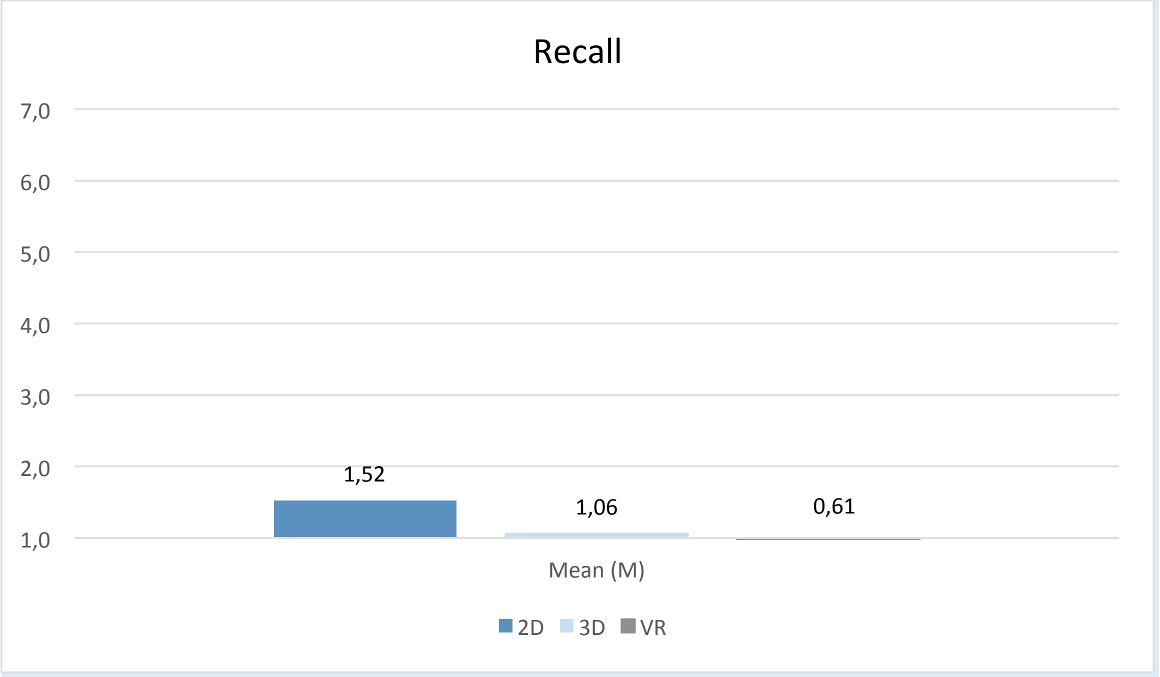

Figure S5. Memory for the placed brands (Recognition: F=14.571, p<.01)

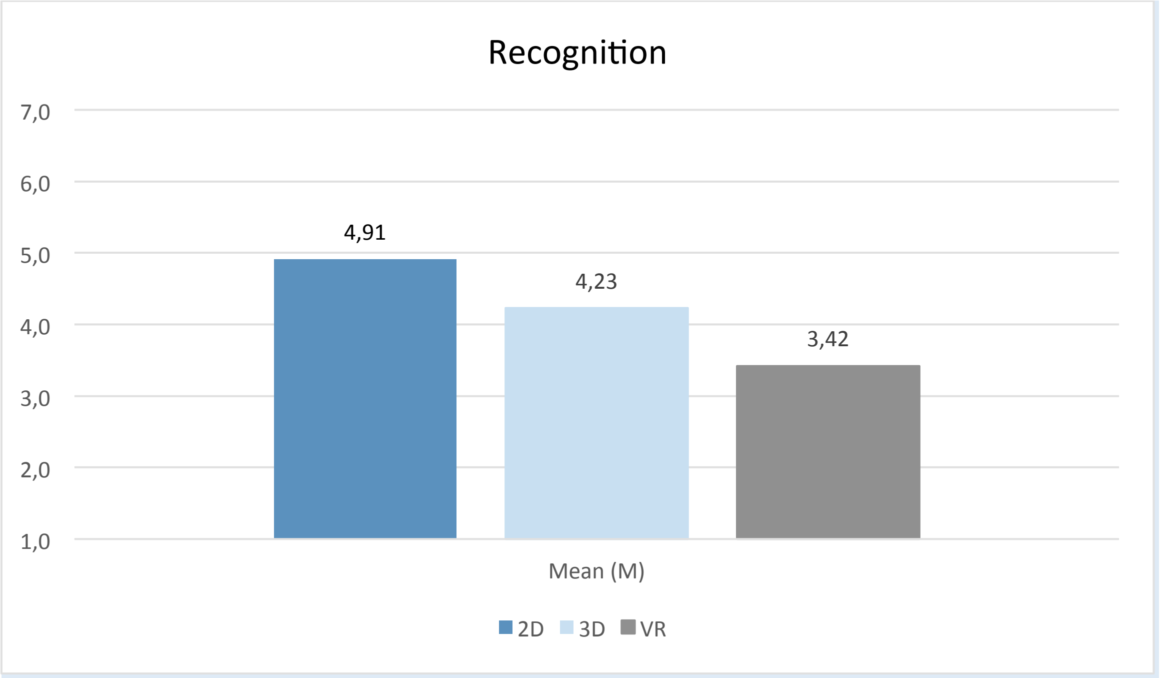

Supplement: S3 Appendix — (PDF) [file pone.0200724.s003.pdf]
